# Rapid extirpation of a North American frog coincides with an increase in fungal pathogen prevalence: Historical analysis and implications for reintroduction

**DOI:** 10.1002/ece3.3468

**Published:** 2017-10-25

**Authors:** Andrea J. Adams, Allan P. Pessier, Cheryl J. Briggs

**Affiliations:** ^1^ Department of Ecology, Evolution and Marine Biology University of California, Santa Barbara Santa Barbara CA USA; ^2^ Department of Veterinary Microbiology and Pathology College of Veterinary Medicine Washington State University Pullman WA USA

**Keywords:** amphibian decline, *Batrachochytrium dendrobatidis*, biodiversity, historical ecology, local ecological knowledge, oral history, reintroduction

## Abstract

As extinctions continue across the globe, conservation biologists are turning to species reintroduction programs as one optimistic tool for addressing the biodiversity crisis. For repatriation to become a viable strategy, fundamental prerequisites include determining the causes of declines and assessing whether the causes persist in the environment. Invasive species—especially pathogens—are an increasingly significant factor contributing to biodiversity loss. We hypothesized that *Batrachochytrium dendrobatidis* (Bd), the causative agent of the deadly amphibian disease chytridiomycosis, was important in the rapid (<10 years) localized extirpation of a North American frog (*Rana boylii*) and that Bd remains widespread among extant amphibians in the region of extirpation. We used an interdisciplinary approach, combining interviews with herpetological experts, analysis of archived field notes and museum specimen collections, and field sampling of the extant amphibian assemblage to examine (1) historical relative abundance of *R. boylii*; (2) potential causes of *R. boylii* declines; and (3) historical and contemporary prevalence of Bd. We found that *R. boylii* were relatively abundant prior to their rapid extirpation, and an increase in Bd prevalence coincided with *R. boylii* declines during a time of rapid change in the region, wherein backcountry recreation, urban development, and the amphibian pet trade were all on the rise. In addition, extreme flooding during the winter of 1969 coincided with localized extirpations in *R. boylii* populations observed by interview respondents. We conclude that Bd likely played an important role in the rapid extirpation of *R. boylii* from southern California and that multiple natural and anthropogenic factors may have worked in concert to make this possible in a relatively short period of time. This study emphasizes the importance of recognizing historical ecological contexts in making future management and reintroduction decisions.

## 1 INTRODUCTION

Conservation biology is gradually transitioning from a discipline of crisis to one of hope. In an era of “crisis fatigue” (Redford & Sanjayan, 2003), reintroduction programs provide much‐needed optimism for recovery (Redford, Aune, & Plumb, 2016). With approximately one‐third of species threatened with extinction, amphibians are the most vulnerable vertebrate group, and creative tools are urgently needed to stem their declines (Kissel, Palen, & Govindarajulu, 2017; Stuart et al., 2004). Amphibian ecologists are beginning to reverse catastrophic losses of some species through reintroductions (Griffiths & Pavajeau, 2008). The spread of non‐native and invasive species, including pathogens, remains a pressing problem, and a critical element in the viability of reintroduction as a conservation strategy is an understanding of the factors causing the geographic emergence and expansion of novel pathogens. Reconstructing historical pathogen distributions and emergence can inform predictions of future pathogen trajectories and the development of reintroduction programs where pathogens have driven localized species extinctions (Kauffman & Jules, 2006; Sainsbury & Vaughan‐Higgins, 2012).

For decades, many amphibian declines remained enigmatic. *Batrachochytrium dendrobatidis* (Bd), the fungal pathogen responsible for the amphibian disease chytridiomycosis, was not identified and described as a causative factor until the late 20th century, owing in part to its unique role, within the phylum Chytridiomycota, as a pathogen of vertebrates (Berger et al., 1998; Longcore, Pessier, & Nichols, 1999). Since then, chytridiomycosis has been implicated in declines or extinctions of hundreds of amphibian species globally (Berger et al., 1998; Fisher et al., 2012; Skerratt et al., 2007). In general, it is rare for disease to be the sole cause of species extinctions (Smith, Sax, & Lafferty, 2006), and until the discovery of chytridiomycosis, disease was largely overlooked as a primary conservation concern in amphibians, as the pathogens known to cause severe declines and extinctions were limited (Collins, Crump, & Lovejoy, 2009). Many theoretic models predict that host populations cannot be driven to extinction by disease alone (Anderson, May, & Anderson, 1992), although host extinction can occur if a reservoir for the pathogen exists, and pathogens can amplify the extinction risk of small host populations (Briggs, Knapp, & Vredenburg, 2010; De Castro & Bolker, 2005). As a result, initially, scientists were hesitant to attribute widespread amphibian declines to epidemic disease (Alford & Richards, 1997; Hero & Gillespie, 1997). Lack of historical information on pathogens may also explain why infectious disease has not traditionally been regarded as an important driver of extinctions (Smith et al., 2006).

Historical ecological knowledge and information, combined with interpretation of ecological change, can make important contributions to informed decision making and addressing contemporary conservation issues (Muths et al., 2016; Swetnam, Allen, & Betancourt, 1999). Diverse historical resources can be drawn upon to measure environmental change, from interviews (Golden, Naisilsisili, Ligairi, & Drew, 2014; Jennings & Hayes, 1994b) to specimens (Lips, 2011), and even works of art (Zerefos et al., 2013). Erroneous perceptions that contemporary conditions are the most accurate standard for ecological health, resulting from a lack of intergenerational communication, lapses in human memory, or the imperceptibly slow deterioration of ecosystems—a phenomenon referred to as shifting baseline syndrome—can lead to inaccurate assumptions about historical abundances and trends and result in poorly informed management decisions (Papworth, Rist, Coad, & Milner‐Gulland, 2009; Pauly, 1995). Bringing historical ecological information to the forefront of contemporary ecological analysis can help guard against this.

**Figure 1:**
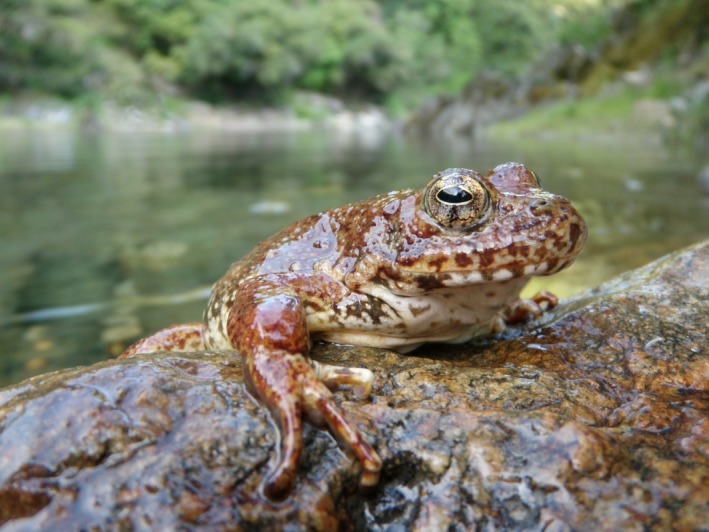
Foothill yellow‐legged frog, *Rana boylii*, from an extant population in northern California. Photograph courtesy of Alessandro Catenazzi

Here, we combined historical and contemporary approaches to determine the timing of extirpation of the foothill yellow‐legged frog, *Rana boylii* (Figure 1), from southern California, USA, and examine its potential causes. *R. boylii* exemplifies many amphibian species in western North America and around the world that experienced marked declines beginning in the 1970s (Berger et al., 2016; Blaustein & Wake, 1990; Corn, 2000; Green & Kagarise Sherman, 2001; Houlahan, Findlay, Schmidt, Meyer, & Kuzmin, 2000; Lips, Mendelson, Muñoz‐Alonso, Canseco‐Márquez, & Mulcahy, 2004; Whitfield et al., 2007). With *R*. *boylii* extirpated from much of its range, ecologists have begun planning for future reintroduction efforts (Lind, 2005; Lind, Spinks, Fellers, & Shaffer, 2011), yet no previous studies have specifically examined the potential role of disease in its decline. In evaluating the relevance and potential success of a reintroduction program, a comprehensive understanding of the original causes of the decline is essential to ensure that the threats have been adequately ameliorated, and to most efficiently and effectively allocate often‐scarce conservation resources (IUCN, 2013).

Often, for amphibian species that have been extirpated from relatively pristine environments, the best‐supported evidence suggests that chytridiomycosis was a significant factor (Berger et al., 1998; Rachowicz et al., 2006; Skerratt et al., 2007). Because the southern California *R*. *boylii* habitats were relatively remote and unaltered at the time declines occurred, we hypothesized that chytridiomycosis was an important causative factor in *R*. *boylii*'s rapid extirpation from the region. To test this hypothesis, we sampled for Bd in archived museum collections and in live amphibians in the field and combined these data with field notes and interviews with experts. We also aimed to determine how quickly the species was extirpated in order to compare trends in *R*. *boylii* occupancy with the timing of Bd emergence. Species are often at more risk of extinction from disease once they have been negatively affected by non‐disease‐related factors (Heard et al., 2013), so we also interviewed experts to examine other threats that may have contributed to declines. Combining these different data types, we expected to find rapid *R*. *boylii* declines coincident with Bd emergence and spread in the region. This study differs from the results of previous work in the *R*. *boylii* decline literature (Davidson, 2004; Davidson, Shaffer, & Jennings, 2002; Kupferberg et al., 2012; Lind, 2005; Lind, Welsh, & Wilson, 1996) by both specifically examining disease as a causative population‐level factor and focusing on southern California—the region from which the species was most rapidly extirpated and for which there is very limited information as a result.

## 2 MATERIALS AND METHODS

### 2.1 Study species

*Rana boylii* (Figure 1) is largely a stream‐dwelling and obligate stream‐breeding species that can inhabit a wide range of fluvial habitats, from first‐ to seventh‐order streams within a single watershed (Bury & Sisk, 1997). Historically they ranged from southern Oregon to Los Angeles County, California, USA, with an isolated population in the Sierra San Pedro Martir in Baja California, Mexico, and from near sea level to 1,219 m (Stewart, Jennings, & Robert, 2005). Listed as a candidate under the California Endangered Species Act and proposed for federal Endangered Species Act listing (U.S. Fish and Wildlife Service, 2015), *R*. *boylii* is absent from approximately half of its formerly occupied sites, with the most pronounced declines and local extirpations concentrated in northern Oregon, southern California, and Baja California, although the extent of disappearances from historical localities may be underestimated (Davidson, 2004; Hayes, Wheeler, Lind, Green, & Macfarlane, 2016; Lind, 2005). Most *R*. *boylii* populations in southern California were apparently stable until the mid‐1970s, when populations collapsed (Jennings, 2005; Jennings & Hayes, 1994a). *R*. *boylii* persistence at historical sites has been positively associated with latitude and precipitation and negatively associated with both upwind and surrounding agriculture (Davidson et al., 2002). Elsewhere in the range, dams and diversions that change flow and thermal regimes have been identified as primary causes of *R*. *boylii* declines (Catenazzi & Kupferberg, 2017; Hayes et al., 2016); however, in southern California, streams that supported the species either were largely undammed or, where dams existed, *R*. *boylii* had persisted for decades at high abundances despite flow regulation.

Until recently, little was known about the susceptibility of *R*. *boylii* to Bd. Experimental infections in the laboratory resulted in either mortality of Bd‐positive individuals not significantly different from those of controls (Davidson et al., 2003) or reduced growth in Bd‐positive juveniles but no mortality (Davidson et al., 2007). In a repeat experiment of Davidson et al. (2007), 16 *R*. *boylii* from the same source population exposed to the same Bd isolate experienced 100% mortality, while no individuals in the non‐Bd treatments (*n* = 16) died (C. Davidson, unpublished data). In 2013, a central California population of *R*. *boylii* experienced a chytridiomycosis‐induced mortality event (Adams et al., 2017), the first such record for this species in the wild.

### 2.2 Interviews and field notes

We used interviews and field notes to determine pre‐extirpation *R*. *boylii* abundance and to assess the rapidity with which the species disappeared from the southern California region. We conducted semi‐directive interviews, which enable the interviewee to guide the conversation beyond a few basic questions, and for which there is no time limit. Semi‐directive interviews are powerful tools for accessing and documenting ecological knowledge that is not available from any other source (Huntington, 1998).

To identify candidate interviewees, we used VertNet (National Science Foundation, 2016) records to identify the collectors of southern California *R*. *boylii* specimens. We identified additional interviewees through conversations with these and other experts in the herpetological and California natural history fields. Candidate interviewees were contacted via email, except for a few who were contacted through Field Herp Forum (Herp Nation Media, 2016) when no email address was readily available. If they consented to an interview, respondents were given an information sheet and oral history interview agreement. The information sheet formally invited the interviewee to participate in the study, briefly described the objective of the study, explained why they were selected as a participant, and explained that participation was entirely voluntary. The interview agreement provided for formal consent to the interview and gave options (via checkboxes) for whether interviewees assented to recording, transcription, and archiving. These instruments were reviewed and approved by the University of California Santa Barbara Human Subjects Committee (protocol # 1‐14‐0245). Interviews took place in person, over the telephone, or via email. We asked a series of general questions about the interviewees’ herpetological expertise, observations of amphibian mortality events and declines, observations of *R*. *boylii* natural history and abundance, and perceived causes of amphibian declines and *R*. *boylii* extirpation (Appendix S1).

When field notes accompanied specimens or observations, we reviewed them for *R*. *boylii* abundance information. A collection of field notes was accessed online through the Museum of Vertebrate Zoology EcoReader (Museum of Vertebrate Zoology, 2016) and the California Academy of Sciences Herpetology Collection Database (California Academy of Sciences, 2016); others were reviewed at natural history museums where they were archived or were provided directly by interviewees. We transcribed responses to interview questions and relative abundance information from field notes and created a database for later analysis. We also used the number of *R*. *boylii* specimens in collections to estimate minimum abundance. To account for the differences between the reliability of primary sources (i.e., specimen collections and field notes) versus interviews that rely solely on memory (Alagona, Sandlos, & Wiersma, 2012), we coded interviews, field notes, and specimen collections as separate information categories.

### 2.3 Museum specimen sampling and analysis

Retrospective surveys of museum specimens can reveal signatures of chytridiomycosis effects on populations, even when observations of die‐offs and mass mortality are lacking (Burrowes, Joglar, & Green, 2004). We sampled postmetamorphic amphibian specimens and used quantitative PCR (qPCR) to detect Bd DNA in order to determine infection status. To identify desired specimens, we searched for species and county records in VertNet (National Science Foundation, 2016) and Specify (University of California Santa Barbara, 2016) and via interviews. In addition to *R*. *boylii* specimens from the southern California study area (defined as Ventura, Santa Barbara, San Luis Obispo, and Los Angeles counties), and because few *R*. *boylii* were collected relative to other species, we sampled all amphibian species that occupied the same streams as *R*. *boylii* in the region: *Anaxyrus boreas halophilus* (California toad); *Anaxyrus californicus* (arroyo toad); *Rana catesbeiana* (bullfrog—a non‐native species that now occupies some former *R*. *boylii* sites); *Rana draytonii* (California red‐legged frog); *Hyliola cadaverina* (California tree frog); *Hyliola regilla* (Pacific chorus frog); and *Taricha torosa* (California newt). We considered specimens desirable if they were one of the species listed above and occurred within the same stream, or the next stream order above or below the same stream as *R*. *boylii,* prior to extirpation.

Specimen sampling, DNA extraction using Macherey‐Nagel DNA FFPE, and qPCR protocols followed Adams et al. (2015), with the exception that 10 μl of extract was used per qPCR reaction. All samples were run in triplicate, with sample replicates run on separate qPCR plates. Specimens were considered positive when two or more samples exhibited qPCR amplification (i.e., as evidenced by an exponential amplification curve). In addition, 57 *T. torosa* specimens were tested for both Bd and *Batrachochytrium salamandrivorans* (Bsal; a fungal pathogen that has led to die‐offs of salamanders in Europe, but which has not yet been detected in the United States (Bsal Task Force, 2016, Martel et al., 2014, 2013)) using duplex PCR following Blooi et al. (2013). These specimens were sampled and DNA extracted as described above.

In addition to qPCR, we conducted histology on select specimens to examine chytridiomycosis status, following Reeder, Pessier, and Vredenburg (2012). This allowed us to confirm the Bd status of 13 specimens collected prior to 1970 that tested positive for Bd via qPCR (i.e., ≥2 amplified replicates in the sample). We also conducted histological examination on six *R*. *boylii* specimens collected from Clear Creek, San Benito County, California, in 1989 that died in captivity at a university laboratory within 6 weeks of their collection from the wild, and an *A. californicus* metamorph from Santa Barbara County, California, that also died in the same laboratory during that time.

### 2.4 Museum specimen statistical analysis

To test various hypotheses for Bd infection in museum specimens, we used an information‐theoretic approach and generalized linear models (GLM). The GLM included a binomial (Bd presence/absence) response and a logit‐link function. If any individuals collected from a site on a particular date were positive (≥2 amplified qPCR replicates), we declared the site positive on that date; if not, we then considered that site–date combination negative for the purposes of the GLM. When multiple species were collected on the same day at the same site, we treated individual species independently. We sampled 1,561 museum specimens for Bd and used 632 site–date–species combinations for the GLM analysis.

Based on other studies of historical Bd prevalence in California (Padgett‐Flohr & Hopkins, 2009; Sette, Vredenburg, & Zink, 2015) and our hypothesis that Bd was a causative factor in *R*. *boylii*'s extirpation in the middle of the 20th century, we expected temporal and spatial variables to be significant predictors of Bd infection in museum specimens. Therefore, we included decade, year, month, 20‐year time interval, and county as categorical predictor variables in the GLM (Table 1). Because temporal variables were correlated, we included only one temporal variable in each model at one time. We also created a spatial variable—distance from earliest positive specimen detected—which enabled us to test the hypothesis that Bd could have spread spatiotemporally from an early Bd introduction (Table 1). Distance from first positive specimen was calculated, in kilometers, from latitude and longitude in decimal degrees using the Vincenty formula for ellipsoidal geodesic distances. As Bd susceptibility can be highly variable between taxa (Searle et al., 2011), we also included two biological variables—family and species—to determine whether there were taxonomic trends in historical Bd status.

**Table 1:** Variables used in generalized linear models used to determine the best predictors of Bd presence/absence in museum specimens

Covariate	Type	Range or levels	Description
Decade	Temporal	1910; 1920; 1930; 1940; 1950; 1960; 1970; 1980; 1990; 2000	Decade that specimen was collected from the wild
20‐year interval	Temporal	1910–1929; 1930–1949; 1950–1969; 1970–1989; 1990–2009	20‐year period that specimen was collected from the wild
Year collected	Temporal	1911–2009	Year that specimen was collected from the wild
Distance from first positive specimen	Spatiotemporal	0–309.9 km	Vincenty distance from the earliest Bd positive detected in this study (z‐transformed)
Month collected	Temporal	1–12	Month of the year that specimen was collected from the wild
County	Spatial	Los Angeles; San Luis Obispo; Santa Barbara; Ventura	California County where specimen was collected from the wild
Species	Biological	*Anaxyrus boreas*;* Anaxyrus californicus*;* Rana catesbeiana*;* Hyliola cadaverina*,* Hyliola regilla*,* Rana boylii*,* Rana draytonii*,* Taricha torosa*	Species of specimen sampled
Family	Biological	Bufonidae, Hylidae, Ranidae, Salamandridae	Family to which specimen sampled belongs

We conducted all analyses using R (R Core Team, 2016) and used a forward selection procedure to determine the predictor variables that best fit the data. To check for multicollinearity among predictors, we used “vif” function in package “car” to ensure that variance inflation factor values were <3 in candidate models that contained more than one predictor (Zuur, Ieno, & Elphick, 2010). Adding predictor variables sequentially in the order presented in Table 1, we used likelihood ratio tests to compare nested models during the forward selection procedure. After completing the forward selection process, we then ranked candidate models according to Akaike's information criterion (AIC) to determine the relative importance of predictor variables. The models with the lowest AIC were considered the best‐supported models by the data, and models with a ∆AIC >2 as compared to the model with the lowest AIC were considered not as well‐supported by the data (Burnham & Anderson, 2004). We used the “allEffects” function from the “effects” package to extract marginalized fixed effects from the model that was the best fit to the data as determined by AIC. We used the “glht” function in the “multcomp” package to conduct Tukey multiple‐comparisons tests to compare the effects differences among levels of the predictor variable in the best‐fit model.

### 2.5 Field sampling

To characterize the current status of Bd in extant stream‐dwelling amphibians within the former range of *R*. *boylii*, we conducted field sampling from 2011 to 2014 in formerly *R*. *boylii*‐occupied habitats in Ventura, Santa Barbara, and Los Angeles counties. All samples were collected in adherence to protocols approved by the University of California, Santa Barbara Institutional Animal Care and Use Committee (Protocol # 825). We sampled all species described above for specimen sampling except for *T. torosa*, because very few individuals of this species were encountered during the nocturnal surveys. Bd sampling and qPCR protocols for field‐collected swabs followed Hyatt et al. (2007) and Boyle, Boyle, Olsen, Morgan, and Hyatt (2004), respectively.

## 3 RESULTS

### 3.1 Interviews

We contacted 29 candidate interviewees and conducted interviews with 21 of them. Of the respondents, four communicated exclusively via email; the remainder were interviewed over the phone or in person. Average interviewee age was 69, and respondents collectively represented 873 years (mean 42 years) of herpetological field experience. Mean verbal interview duration was 1.2 hr.

#### 3.1.1 Reasons for *R. boylii* decline

Most respondents declined to speculate what could have caused *R*. *boylii* extirpations from southern California. Responses centered around several common hypotheses of amphibian declines, including chytridiomycosis, pet trade, exotic species, and climate change (Catenazzi, 2015); however, two relatively unique threats emerged: increased recreational use of habitats and extreme flooding (Table 2). Three respondents reported observing the localized extirpation of once‐abundant *R*. *boylii* populations after extreme flood events in California: (1) Tulare County in the late 1960s (Interview 9); (2) Caliente Creek (Kern County) in the mid‐1970s (Interview 11); and (3) Evey Canyon in the San Gabriel Mountains (Los Angeles County) in the late 1960s (Interview 9). The Evey Canyon site shifted from having a very abundant population to no *R*. *boylii* found, according to the interviewee:

**Table 2:** Interviewee responses to inquiry of potential causes of *R*. *boylii* extirpation from southern California

Cause	Years^a^	Interview #	Explanation
Amphibian pet trade and exotic amphibians	175 [58]	10, 12, 20	Amphibians wild‐caught; availability in local department stores; pet releases into the wild
Bd	195 [49]	3, 10, 12, 20	Chytridiomycosis
Bd + climate change	54	10	Climate change exacerbates conditions when Bd is already present, making amphibians more susceptible to disease
Bd + fish stocking	56	12	Releases Bd zoospores into new waterways
Bd + flooding	56	12	Unusual frequency of rain events & overcast days reduces opportunities for frog basking (higher temperatures can mitigate chytridiomycosis)
Climate change	54	10	Declines trend from south to north; declines occurred earlier in drier climates
Drought	60	4	1955–1960 and 1971–1978 were very dry years
Non‐native fish	63	17	Non‐native fish introduced, resulting in predation
Flooding (1968–1969)	210 [53]	9, 11, 12, 19, 20	Extreme flood events scoured frogs to lower elevations/less suitable habitat; permanent change in habitat quality and suitability
Recreation	234 [59]	12, 17, 19, 20	Increased and intensified recreational uses of streams

a Cumulative years of herpetological experience represented by respondents; number in brackets shows average number of years.

The frogs [*R. boylii*] were extremely abundant there…you'd walk along the creek for a couple hundred yards and collect a sample of 15 or 20…the frogs were just all over the place. But after the ‘69 floods…I went back in ‘70…there weren't frogs in that creek at all and there haven't been since. (Interview 9)

#### 3.1.2 *R*. *boylii* occurrence and abundance in southern California

Despite the number of naturalists frequenting various portions of the study area, none were able to produce a record of *R*. *boylii* from later than 1977. One interviewee confirmed his sighting of one *R*. *boylii* individual in Piru Creek, Los Angeles County, on 6 July 1977, which is the last known record of the species in southern California (Hayes et al., 2016; Jennings & Hayes, 1994a). Extensive *R*. *boylii* resurveys were conducted in southern California by two independent research teams from 1981 to 1993 and from 1988 to 1991; however, none encountered the species. In addition, we did not encounter the species during our field surveys from 2011 to 2014. Several *R*. *boylii* collections were made in the 1940s and 1950s, along with field notes containing abundance information such as “very common” (R. Zweifel field notes, 20 March 1948) and “fairly abundant” (Interviewee 20 field notes, 3 May 1970); only one field note entry was found that reported a negative survey for *R*. *boylii* prior to the late 1970s (R. Zweifel field notes, 2 April 1950). Some interviewees reported abundance information such as “15‐20 individuals per mile of stream” (Interview 8).

### 3.2 Chytridiomycosis in *Rana boylii*

Interviews revealed two previously unknown *R*. *boylii* mortality events attributable to chytridiomycosis, both outside of the immediate study area. In 1989, six live *R*. *boylii* individuals collected from Clear Creek, San Benito County (central California), were taken to a university laboratory where they were housed in a 60‐gallon tank with recirculating water. Within 6 weeks, all frogs succumbed to an unknown infection, which we confirmed to be chytridiomycosis (see the Section “3.1.2” below). Histological examination of these frogs revealed hyperkeratosis and hyperplasia in all six animals, consistent with chytridiomycosis, and Bd organisms in one (Table 3). Necropsy conducted on one of the individuals suggests that this animal died of chytridiomycosis. These animals appeared healthy when collected from the stream where they occurred, although they were taken from a stream that was actively being used by off‐road vehicles (Interview 19). It is unknown whether they contracted Bd from the field or in the laboratory, as this event occurred a decade before Bd was described (Longcore et al., 1999). The second mortality event was a *R*. *boylii* die‐off in a stream in Stanislaus County (central California) in 1986. Approximately 85 dead individuals were observed over a period of 2 weeks, until there were only a few animals left (Interview 12). Retrospective histology on these specimens confirmed that they were infected with Bd and showed symptoms of chytridiomycosis (Interviews 10 and 12). Although in a part of California where *R*. *boylii* is extant, the *R*. *boylii* population at this locality has not recovered to the level of abundance present prior to this chytridiomycosis outbreak (Interview 12).

**Table 3:** Results of histological examination of formalin‐fixed, ethanol‐preserved museum specimens

Institution	Collection #	Species	County	Year	qPCR replicates positive	Histology result
CAS	39865	*Anaxyrus boreas*	Los Angeles	1915	2	Lesions of hyperplasia and hyperkeratosis, no Bd organisms
CAS	39867	*A. boreas*	Los Angeles	1915	3	Lesions of hyperplasia and hyperkeratosis, no Bd organisms
CAS	63049	*Hyliola cadaverina*	Ventura	1927	2	Negative
MVZ	27881	*Rana catesbeiana*	Los Angeles	1939	3	Negative
MVZ	33668	*Rana boylii*	Ventura	1940	2	Negative
MVZ	33672	*R. boylii*	Ventura	1940	2	Negative
LACM	13455	*Rana draytonii*	Los Angeles	1947	2	Negative
LACM	13703	*R. boylii*	Ventura	1954	2	Negative
LACM	13496	*R. draytonii*	Ventura	1954	2	Negative
CAS	181067	*A. boreas*	Santa Barbara	1961	2	Negative
LACM	13457	*R. draytonii*	Los Angeles	1963	2	Negative
CAS	190944	*Anaxyrus californicus*	Santa Barbara	1966	2	Negative
LACM	76274	*R. draytonii*	Ventura	1968	3	Negative
Private	NA	*A. californicus*	Santa Barbara	1989	1	Hyperplasia and hyperkeratosis, no Bd organisms
Private	NA	*R. boylii*	San Benito	1989	0	Hyperplasia and hyperkeratosis with Bd organisms
Private	NA	*R. boylii*	San Benito	1989	0	Hyperplasia and hyperkeratosis, no Bd organisms
Private	NA	*R. boylii*	San Benito	1989	0	Hyperplasia and hyperkeratosis, no Bd organisms
Private	NA	*R. boylii*	San Benito	1989	0	Hyperplasia and hyperkeratosis, no Bd organisms
Private	NA	*R. boylii*	San Benito	1989	0	Hyperplasia and hyperkeratosis, no Bd organisms
Private	NA	*R. boylii*	San Benito	1989	0	Hyperplasia and hyperkeratosis, no Bd organisms

### 3.3 Museum specimens

**Figure 2:**
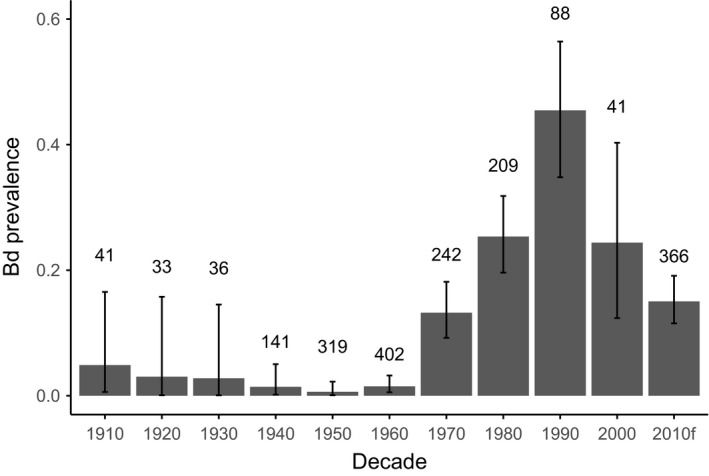
Bd prevalence through time for all museum specimens sampled. Each bar represents a decade that begins with the year indicated by the *x*‐axis label. Error bars represent Clopper–Pearson binomial confidence intervals. Numbers above the bars denote sample size. Data are from specimens collected from the study area, including Los Angeles, Ventura, and San Luis Obispo, and Santa Barbara counties. The “2010f” category represents Bd samples collected from live postmetamorphic amphibians sampled in the field from 2011 to 2014

**Figure 3:**
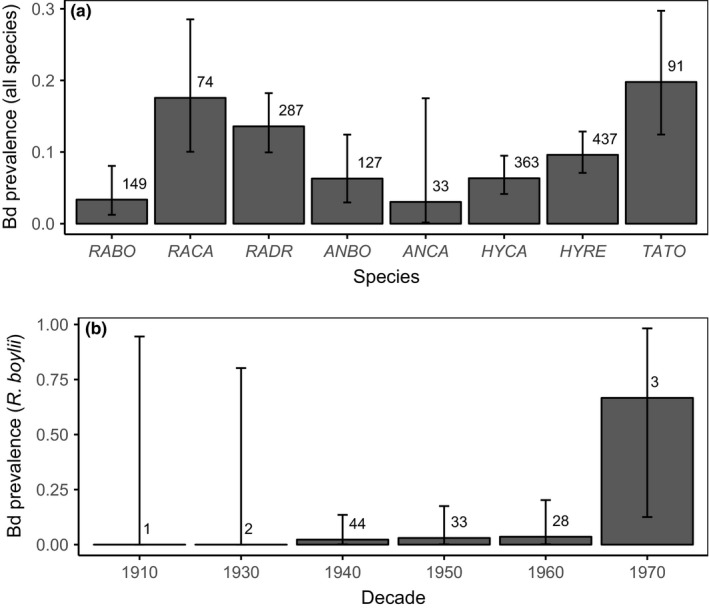
Bd prevalence of museum specimens, representing (a) all species; and (b) *R*. *boylii* only, by decade. Species codes: *RABO *= *Rana boylii*;*RACA *= *Rana catesbeiana*;*RADR *= *Rana draytonii*;*ANBO *= *Anaxyrus boreas*;*ANCA *= *Anaxyrus californicus*;*HYCA *= *Hyliola cadaverina*;*HYRE *= *Hyliola regilla*;*TATO *= *Taricha torosa*. Decades are indicated on the *x*‐axis in (b) by the first year in each decade

**Figure 4:**
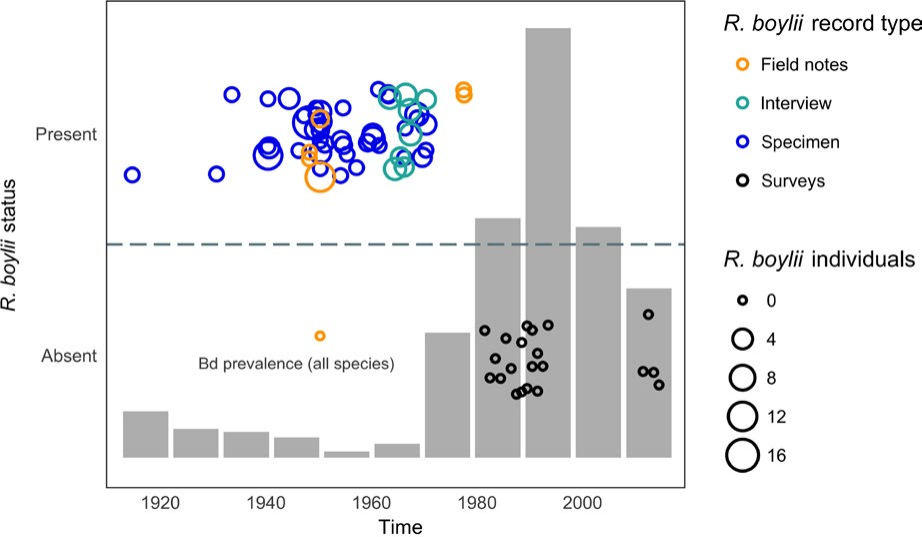
*Rana boylii* relative abundance (jittered circles) by record type through time. Specimen abundances are represented by the number of postmetamorphic individuals collected at a site on a particular date. Background plot (histogram in gray) shows relative Bd prevalence of all species detected from museum specimens through time (see Figure 2 for details). For Bd prevalence through time in *R*. *boylii*, refer to Figure 3b

We sampled 1,561 museum specimens from eight institutional collections (Appendix S2). All species tested positive for Bd, and overall infection prevalence was 10.6% (149 of 1,561 samples). Bd‐positive individuals were present in all counties sampled. The earliest Bd positives detected with qPCR were two *A. boreas* specimens collected from Los Angeles County in 1915. Bd prevalence varied temporally and showed a steady increase beginning in the 1970s and continuing through the 1990s, followed by a decline in prevalence after the 1990s (Figure 2). California newts (*T. torosa*) and non‐native American bullfrogs (*R. catesbeiana*) had the highest prevalence of infection among species (Figure 3a). Bd prevalence in *R*. *boylii* was among the lowest (with higher prevalence in the 1970s); however, samples from this species were limited to the period prior to its extirpation, with prevalence in other species increasing during the 1990s, by which time *R*. *boylii* was extirpated from the study area (Figures 3b and 4).

**Figure 5:**
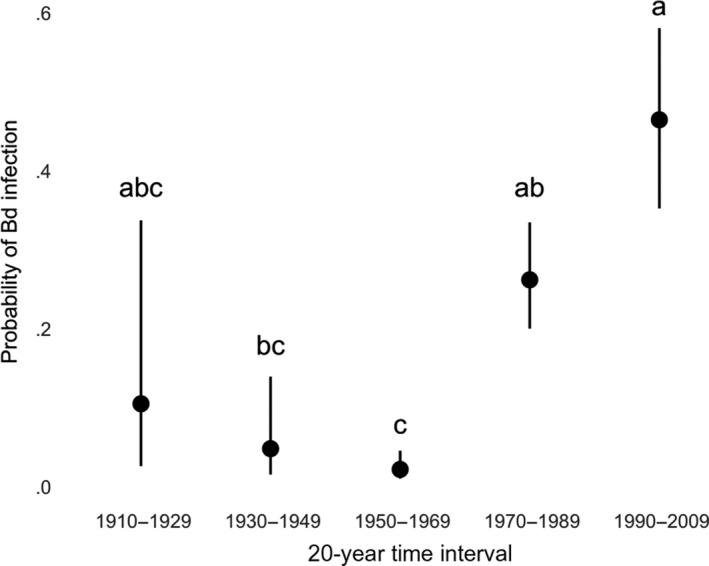
Probability of Bd infection for 20‐year time intervals as indicated by the best‐fit generalized linear model of museum specimen samples. Error bars indicate 95% confidence intervals. Letters above the bars indicate Tukey groups for 20‐year time intervals from the best‐fitting model of Bd presence/absence in all species: Time intervals that do not share a letter are significantly different from one another

**Figure 6:**
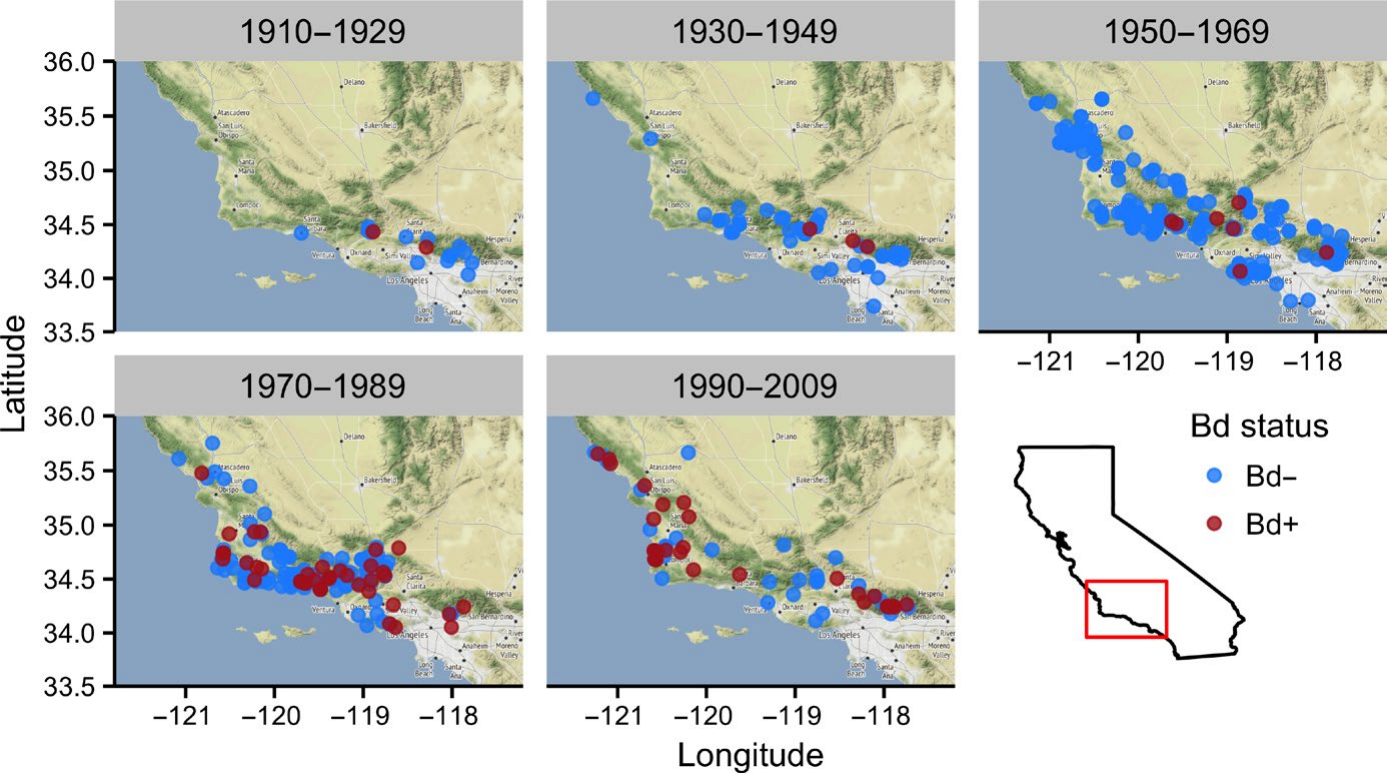
Map of localities sampled for Bd in museum specimens from 1915 to 2009 as used in the generalized linear model. Dates above each map represent the 20‐year time interval in which each specimen was collected. Data were arranged prior to mapping so that Bd‐positive samples would be superimposed on Bd‐negative samples when they overlapped. Data include sampled specimens collected from Ventura, Santa Barbara, Los Angeles, and San Luis Obispo counties

The GLM analysis indicated that Bd presence/absence in museum specimens was best predicted by 20‐year time interval and distance from the first positive specimen (Table 4 and Figure 5), suggesting that Bd spread spatiotemporally from the area that is now the greater Los Angeles area in the early 1900s (Figure 6). Species, month, year, decade, family, and county were not important predictors in the GLM. As species was not an important predictor, the intrinsic confounding of species and time, due to historically uneven specimen collection, likely did not drive the pattern we observed. The importance of 20‐year time interval despite the relative unimportance of decade indicates that longer time intervals are required to observe a clear trend in the independent samples we collected.

**Table 4:** Candidate generalized linear models used to determine the best predictors of Bd presence/absence in museum specimens

Rank	Model	*K*	logLik	AIC_c_	∆AIC_c_	AIC_w_
1	20‐year interval	5	−195.39	400.87	0	0.49
2	20‐year interval + Distance from first positive specimen	6	−195.11	402.36	1.49	0.23
3	20‐year interval + Family	8	−193.37	402.96	2.09	0.17
4	20‐year interval + County	8	−194.53	405.29	4.42	0.05
5	Decade	10	−193.46	407.27	6.4	0.02
6	20‐year interval + Species	12	−191.49	407.48	6.61	0.02
7	20‐year interval + Month	16	−188.61	410.11	9.24	0
8	Year	80	−153.14	489.8	88.93	0
9	Null	1	−255.06	512.13	111.26	0

*K* = Number of parameters in the model.

Histological examination revealed evidence of chytridiomycosis in two of the 13 specimens collected prior to 1970 that were qPCR‐positive for Bd (Table 3)—two *A. boreas* specimens collected from Los Angeles County in 1915 (CAS 39865, 39867). Both individuals had lesions of hyperplasia and hyperkeratosis (a thickened stratum corneum, consistent with chytridiomycosis infection (Pessier, Nichols, Longcore, & Fuller, 1999)). All of the *R*. *boylii* collected from Clear Creek in San Benito County in 1989 that later died in captivity exhibited hyperkeratosis and hyperplasia, as did the *A. californicus* metamorph that died in the same university laboratory at the same time as the *R*. *boylii* collected from Clear Creek. No visceral lesions of other infectious diseases known to cause mortality events of amphibians (e.g., Ranavirus) were observed in any of the animals histologically examined. None of the 59 specimens additionally tested for Bsal were positive for that pathogen, consistent with sampling efforts that have not yet found Bsal infection in North America (Bsal Task Force, 2016).

### 3.4 Field sampling

**Figure 7:**
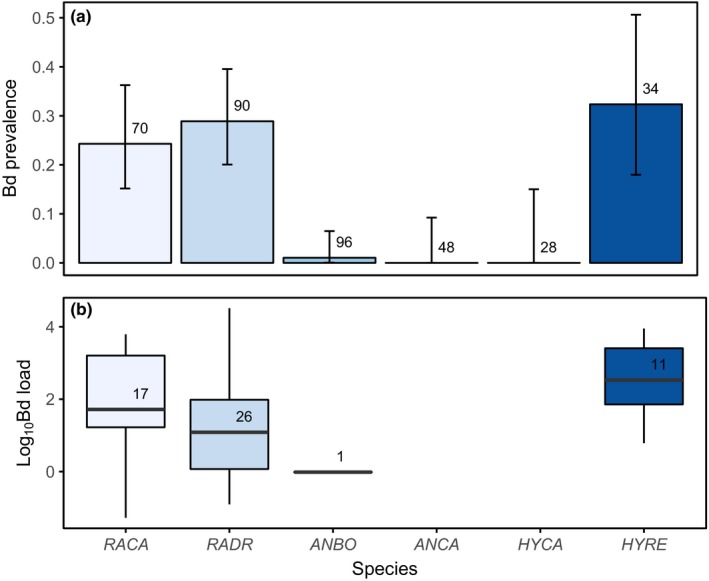
Bd infection among live anurans sampled for Bd in the wild during 2011–2014 within the former range of *Rana boylii* in southern California (Ventura, Santa Barbara, and Los Angeles counties), indicating (a) Bd prevalence; and (b) pathogen load of Bd‐positive individuals. Error bars in (a) represent the 95% Clopper–Pearson binomial confidence intervals. Bold horizontal lines within each boxplot in (b) indicate the median, boxes show the interquartile (IQ) range, and whiskers show the range within 1.5 times the IQ range. Numbers above the bars indicate (a) total sample size for each species or (b) the number of Bd‐positive individuals. *RACA *= *Rana catesbeiana*;*RADR *= *Rana draytonii*;*ANBO *= *Anaxyrus boreas*;*ANCA *= *Anaxyrus californicus*;*HYCA *= *Hyliola cadaverina*;*HYRE *= *Hyliola regilla*

Between 12 March 2011 and 9 September 2014, we captured and sampled 366 postmetamorphic anurans. Only *A. californicus* and *H. cadaverina* did not test positive for Bd; infection prevalence (Figure 7a) for all species combined was 15% (55 of 366 samples). Among species, Bd prevalence and load were highest in the two ranid species sampled (*R. draytonii* and *R. catesbeiana*) and *H. regilla* (Figure 7b).

## 4 DISCUSSION

### 4.1 *Bd* prevalence coincident with *R*. *boylii* extirpation

This study provides a clearer estimate of the timing of *R*. *boylii* extirpation from southern California, focuses the geographic scope of *R*. *boylii* decline analysis to this region, and is the first to examine disease as a causative factor in the historically precipitous declines of this species. One of the most important findings of the current study is that the proliferation of Bd in southern California coincided with the rapid extirpation of *R*. *boylii* from the region (Figures 2 and 4). Many declines ascribed to Bd have been unusually rapid as compared to those attributed to other causes, and in retrospective disease‐related analyses of museum specimens, high Bd prevalence is often observed during and after declines, but not before (Berger et al., 1998; Cheng, Rovito, Wake, & Vredenburg, 2011; Gillespie, Hunter, Berger, & Marantelli, 2015; Lips et al., 2006; Muths, Corn, Pessier, & Green, 2003; Vredenburg, Knapp, Tunstall, & Briggs, 2010). When Bd first arrives in a naïve population of susceptible hosts, infection prevalence rapidly increases, followed by either extirpation or transition to an eventual enzootic steady state of disease dynamics, wherein disease prevalence decreases (Briggs et al., 2010; Vredenburg et al., 2010).

The spatial pattern of *R*. *boylii* decline also mirrors patterns of Bd emergence. The gradual, progressive south‐to‐north trend of *R*. *boylii* declines and extirpations in California was mentioned by two *R*. *boylii* experts during their interviews (9 and 10) and is supported by the literature (Davidson et al., 2002; Lind, 2005). In addition, the wavelike pattern of Bd emergence and proliferation observed in this study (Figure 6) is consistent with observations elsewhere in California (Padgett‐Flohr & Hopkins, 2009; Vredenburg et al., 2010) and other parts of the world (Laurance, McDonald, & Speare, 1996; Lips, Diffendorfer, Mendelson, & Sears, 2008). Abundance information estimated from interviews, specimen collections, and field notes suggests that *R*. *boylii* populations in southern California prior to the proliferation of Bd were not small (Figure 4). While these are only relative abundance estimates and not the results of field surveys specifically targeted for quantifying *R*. *boylii* occupancy, they suggest that the species was relatively abundant shortly before it was extirpated (Figure 4).

### 4.2 Pathogen detection from museum specimens

Because of the large sample sizes in every decade from 1940 to 1980, we can be quite confident that Bd prevalence was initially low and then increased markedly. Because of the smaller sample sizes in the early decades and the failure of histology to detect Bd in early‐collected specimens, we are less confident of the prevalence prior to 1940. The probability of Bd detection from formalin‐fixed, ethanol‐preserved specimens increases as prefixation (live animal) infection load increases (Adams et al., 2015); therefore, it is likely that higher Bd prevalence observed between the 1970s and 1990s in this study also indicates higher pathogen burden, which is consistent with an epizootic disease phase.

It is important to note that due to the difficulty in recovering Bd DNA from formalin‐fixed museum specimens using qPCR (Adams et al., 2015), estimated Bd prevalence from museum specimens will appear lower than is actually the case; therefore, comparing contemporary, field‐based Bd data to specimen Bd data should be conducted with care. Although the prevalence estimates for field and specimen samples collected since the 2000s appear similar (Figures 3a and 7a), prevalences are likely much lower now than they were during the initial epizootic stage from the 1970s through the 1990s, due to better detection of Bd DNA from fresh field samples as compared to formalin‐fixed specimens (Figure 2). In addition, postfixation Bd load does not correlate with prefixation Bd load (Adams et al., 2015; Richards‐Hrdlicka, 2012); therefore, we are not able to infer prefixation (i.e., live animal) loads from the qPCR results we obtained from the specimens.

Despite not being able to detect chytrid organisms via histology in some qPCR‐positive specimens sampled from prior to 1970, we considered these positive for the purposes of this study, based on the following: (1) The two *A. boreas* specimens from 1915 (the earliest positives detected in this study) had lesions consistent with chytridiomycosis despite lacking Bd organisms; (2) histology can produce false negatives even in highly infected individuals due to the patchy or uneven distribution of infection on amphibian skin or low zoosporangia density (Boyle et al., 2004; Pessier et al., 1999; Reeder et al., 2012); (3) we were limited to sampling a single, small patch of skin on each specimen by some lending institutions, so the possibility that we missed sampling infected areas, due to the patchy distribution of chytridiomycosis on amphibian skin, is high; (4) earlier swabbing events may have removed some of the zoosporangia‐containing tissue that is important for histological diagnosis (Fong et al., 2015); (5) when the specimen sampling and qPCR protocols we followed are used, Bd contamination is unlikely (Adams et al., 2015); and (6) considering the pre‐1950s specimens positive based on two qPCR positives was conservative to our hypothesis that Bd prevalence increased in the middle of the 20th century. Items 1–5 above have implications extending beyond this study—we urge curators of natural history collections and the researchers that use them to communicate with one another regarding the specimens that have been sampled for Bd so that subsequent researchers can be aware of the increased risk of false histology negatives after specimens have been sampled, as the act of swabbing may remove zoosporangia‐containing tissue (Adams et al., 2015; Fong et al., 2015).

### 4.3 Reservoir hosts and Bd introduction

The presence of a pathogen reservoir can allow a pathogen to lead to disease‐induced extinction of some host species (Briggs et al., 2010; De Castro & Bolker, 2005). Our 2011–2014 field sampling of extant species within the former range of *R*. *boylii* indicates that Bd prevalence and loads are highest in *R. catesbeiana* (American bullfrog) and *H. regilla* (Figure 7). Both *R. catesbeiana* and *H. regilla* are considered to be vectors of Bd in California (Adams et al., 2017; Padgett‐Flohr & Hopkins, 2009; Reeder et al., 2012), and as an often‐tolerant Bd reservoir host, bullfrogs have been implicated in declines of amphibians in places throughout the world where they are not native, including California (Daszak et al., 2004; Jennings & Hayes, 1994a; Miaud et al., 2016). *H. regilla* and *R. catesbeiana* may have provided sufficient reservoir hosts to allow for Bd persistence in the system, facilitating chytridiomycosis‐induced extinction of *R*. *boylii*. While some laboratory studies have suggested that bullfrogs may not always make suitable reservoir hosts for Bd (Eskew, Worth, Foley, & Todd, 2015; Gervasi et al., 2013), our observation that bullfrogs have the highest Bd prevalence and loads among species sampled in the field (Figure 7), and previous work showing that bullfrog sympatry is an important predictor of Bd infection in *R*. *boylii* (Adams et al., 2017), supports the hypothesis that bullfrogs are a vector of Bd in California, including where *R*. *boylii* are extant.

Bullfrog farming may have played a key role in introducing and spreading Bd in California. Bullfrogs were brought to California in the early 20th century from the eastern United States, where Bd may have occurred in an enzootic state for much longer than in the western United States (Talley, Muletz, Vredenburg, Fleischer, & Lips, 2015). Between 1900 and 1930, several farms that originally imported bullfrogs from the eastern United States were present throughout California, resulting in bullfrog introductions to the wild (Collins et al., 2009). Around 1912, bullfrogs were introduced to an artificial lake near Topanga Canyon (Los Angeles County) from New Orleans, Louisiana, which provided a source population for subsequent introductions throughout California (Jennings & Hayes, 1994b). The earliest Bd positives detected in our study, two *A. boreas* collected from Big Tujunga Canyon (Los Angeles County) in 1915, may have been the result of nearby bullfrog farming operations such as the one in Topanga Canyon. Although Bd appears to have arrived early in California—even predating introductions of the Bd‐tolerant African clawed frog, *Xenopus laevis* (Huss, Huntley, Vredenburg, Johns, & Green, 2013; Weldon, du Preez, Hyatt, Muller, & Speare, 2004)—our results suggest that Bd may have experienced multiple invasions with limited spread (Sette et al., 2015) prior to its proliferation beginning in the 1970s in southern California (Figure 2). In addition, Bd's ubiquity among sites sampled in the 2010s, its relatively low prevalence and pathogen loads among the anuran community, and a postepizootic pattern of decline in prevalence after the 1990s indicate that it may have reached an enzootic state in the study area (Figures 2 and 7).

### 4.4 Anthropogenic stressors

The 1970s extirpation of *R*. *boylii* from southern California is contemporaneous with some of the earliest reports of enigmatic amphibian declines in western North America (Blaustein & Wake, 1990; Drost & Fellers, 1996; Green & Kagarise Sherman, 2001; Sherman & Morton, 1993), Puerto Rico (Burrowes et al., 2004), and Australia (Berger, Speare, & Hyatt, 1999). After World War II, California's human population grew much faster than most other U.S. regions (Hope, 2011), sparking sprawling development and facilitating the spread of exotic pathogens that were once restricted to their native habitats or near ports of entry. Cities and intensive human activity increase opportunities for direct amphibian pathogen transport (Price, Garner, Cunningham, Langton, & Nichols, 2016) and spread via other hosts (Schloegel et al., 2009; Weldon et al., 2004). Roads are often associated with increased pathogen transmission (Jules, Kauffman, Ritts, & Carroll, 2002), and in a retrospective study in northern California, the earliest Bd positives occurred closest to a transportation corridor (De León, Vredenburg, & Piovia‐Scott, 2016), suggesting that roads are able to facilitate the spread of Bd.

Increased human use of natural areas within *R*. *boylii* habitat during the rapid development of southern California in the middle of the 20th century was likely exacerbated by the proliferation and wide availability of the pet trade. At this time, the pet trade was thriving—wild‐caught red‐eared sliders (*Trachemys scripta elegans*) and frogs were widely available at department stores such as Grant's in southern California (Interview 12) (Salisbury Post, 2014). A burgeoning pet trade and increased use of backcountry areas may have contributed to the spread of Bd, due to the increased likelihood of captive releases of pets back into the wild.

Several interviewees cited an increase in human use of habitats immediately prior to *R*. *boylii* extirpations (Table 2). In the two chytridiomycosis‐related *R*. *boylii* mortality events reported in this study, the habitats of both populations were actively being used by off‐road vehicles at the time (Interviews 12 and 19; although the source population for the captive animals that experienced mortality in the laboratory did not succumb to chytridiomycosis in the field). This coincidence suggests that increased recreational use by humans could facilitate the spread and proliferation of Bd in these habitats. In addition, environmental disturbances may stress amphibians enough to suppress their immune defenses against Bd (Rollins‐Smith et al., 2002) (but see Searle, Belden, Du, & Blaustein, 2014).

### 4.5 Extreme flooding

In January and February 1969, extreme flooding caused extensive damage in southern California, killing at least 115 people (U.S. Army Corps of Engineers, 1969). It has been suggested that the 1969 flood events led to the extirpation of *R*. *boylii* from southern California (Sweet, 1983), and five interviewees mentioned flooding as a proximate or ultimate cause of *R*. *boylii* extirpation (Table 2). Although only one of these observations of *R*. *boylii* extirpations following flood events occurred in the southern California study area (Evey Canyon, Los Angeles County), they suggest that the species could be vulnerable to extreme flood events. *R*. *boylii* is a stream obligate and rarely found more than a few meters from water (Kupferberg, 1996; Zweifel, 1955). Radio‐tracked *R*. *boylii* in northern California have been observed up to 40 m from the stream channel during relatively extreme precipitation events, suggesting that they will use uplands to avoid flows that would otherwise sweep them downstream, and will avoid peak flows in the main stream channel by overwintering in tributaries (Bourque, 2008). In another study, the magnitude of winter floods did not affect *R*. *boylii* clutch density (a measure of adult abundance), but dam releases timed asynchronously with environmental cues for flooding caused scouring of clutches and *R*. *boylii* recruitment losses (Kupferberg et al., 2012).

Evey Canyon is a steep site where frogs may not have been able to retreat to uplands during flooding. The 1969 flood event, however, was not the first of its kind in the region. The 1969 floods are considered the most damaging on record because they occurred after extensive development, but flooding in 1907, 1914, 1916, and 1938 may have equaled the 1969 floods in magnitude (U.S. Army Corps of Engineers, 1969). In addition, sediment deposition indicates that the 1969 floods were predated by much larger flood events (Hendy, Napier, & Schimmelmann, 2015; Ingram, 2013). Flooding has the potential to cause catastrophic mortality events, affecting population age structure, but is unlikely, on its own, to cause the rapid extirpation of a species from an entire region (Corn, 1994; Metter, 1968). Therefore, flooding was probably not the sole cause of *R*. *boylii* extirpation from southern California. Instead, we suggest that the flood event occurred at the same time as when Bd prevalence was on the rise (Figure 2), so chytridiomycosis and extreme flooding may have acted in concert to extirpate the species.

### 4.6 Why does *R*. *boylii* persist elsewhere in its range?

Regional contrasts in phylogeography, geomorphology, and climate may contribute to the absence of *R*. *boylii* in southern California relative to northern California and southern Oregon. *R*. *boylii* is more phylogenetically distinct at the edges of its range than at the core—the most distinct and divergent lineages occur at the southern portion of its range (Lind et al., 2011)—suggesting that the southern California populations may have differed in their susceptibility to Bd and subsequent extirpation. Differential outcomes in Bd susceptibility have been observed in the field (Briggs et al., 2010), and under identical conditions in the laboratory (Searle et al., 2011) (C. Davidson, unpublished data). Immunological and genetic responses to Bd infection may reduce frogs’ Bd susceptibility, resulting in persistent populations that are able to recover if there is a large enough population to sustain itself while this evolution occurs over generations (Knapp et al., 2016; Ramsey, Reinert, Harper, Woodhams, & Rollins‐Smith, 2010; Savage & Zamudio, 2011).

Although the interviews and histological examinations presented here, combined with a field study (Adams et al., 2017), indicate that *R*. *boylii* populations in central and northern California have also been affected by Bd and flooding, differences in environmental context may have contributed to a different outcome. The Mediterranean streams of southern California are qualitatively different from those in northern California. In southern California, highly variable hydrologic regimes are characterized by flashier flows, more ephemeral channels, and a higher degree of intermittency due to the lower total rainfall of the region (Cooper, Lake, Sabater, Melack, & Sabo, 2013). In *R*. *boylii*‐occupied regions of northern California, the greater density of dendritic river networks creates wetter habitats with more tributaries and wider floodplains, making *R*. *boylii* populations less isolated, facilitating dispersal and recolonization of unoccupied streams (Groom, Meffe, & Carroll, 2006; Larned, Datry, Arscott, & Tockner, 2010).

Differences in timing of pathogen emergence may also contribute to amphibian persistence. Bd may not have arrived in central and northern California until later in the 20th century than our observations for southern California: Retrospective surveys of museum specimens suggest that Bd arrived in the San Francisco Bay area of California in the late 1950s (Padgett‐Flohr & Hopkins, 2009) (but see Huss et al., 2013) and as late as the mid‐1970s in parts of northern California (De León et al., 2016). One interviewee (10) reported heavy flooding in northern California in 1956 and 1964, which may have occurred prior to Bd's arrival in that region.

Bd has a low tolerance for warm temperatures, leading many to hypothesize that latitude and altitude are positively correlated with Bd infection (Knapp, Briggs, Smith, & Maurer, 2011; Kriger & Hero, 2008; Kriger, Pereoglou, & Hero, 2007). Under this assumption, one might expect southern California *R*. *boylii* populations to be less susceptible to Bd as compared to those further north. Southern California's Mediterranean climate is characterized by cool, wet winters and warm, dry summers. While the mean temperature is warmer overall as compared to the rest of California, southern California's streams and microhabitats are often within the temperature threshold for Bd's optimal growth (Klose, Cooper, Leydecker, & Kreitler, 2012; Piotrowski, Annis, & Longcore, 2004). In an extant population of *R*. *boylii* in central California, water temperature was not predictive of either Bd prevalence or load in *R*. *boylii*, although low stream flows were predictive (Adams et al., 2017). Therefore, it may be other aspects of climate beyond ambient temperatures (i.e., precipitation, alternation of extreme droughts and floods) that distinguish the southern part of *R*. *boylii*'s range.

Frogs vary in their responses to different strains and isolates of Bd (Piovia‐Scott et al., 2015) and novel strains can cause negative impacts to species at the same time that endemic strains do not (Gahl, Longcore, & Houlahan, 2012). Yet, separate Bd strains do not always result in different Bd outcomes (Knapp et al., 2016). There is no evidence to suggest that the Bd DNA detected in this study is from a strain endemic to the region. All of the wild Bd isolates genotyped to date from California and the Pacific coast of North America are part of the Bd‐GPL (Global Panzootic Lineage), belonging to the rapidly dispersed hypervirulent lineage of Bd, and there is no genetic evidence of any endemic lineages in North America (James et al., 2015). Where endemic and epizootic Bd lineages are sympatric, the endemic Bd shows a much more limited geographic distribution, and Bd‐GPL may be better at dispersing in a heterogeneous landscape as well as infecting a broader range of host species (Jenkinson et al., 2016).

## 5 CONCLUSION

Emerging infectious diseases are a significant threat to global biodiversity, and information on the introduction, spread, and effects of novel pathogens is essential for enacting appropriate responses to this threat (Roy et al., 2016). Anthropogenic forces have probably played an important role in the spread of hypervirulent lineages of Bd globally (Farrer et al., 2011), and the rapid growth of mid‐20th‐century southern California coinciding with increased Bd prevalence and the extirpation of a once‐common anuran is consistent with this hypothesis. This study emphasizes the importance of the human context within which most host–pathogen systems occur, including perceptions of biodiversity loss. Many interviewees were reluctant to hypothesize why *R*. *boylii* disappeared in such a short period of time, in part because amphibian populations naturally fluctuate; many did not know that anything unusual was happening at the time. As one interviewee stated:

…I would go to places I had seen them [*R. boylii*] and they wouldn't be there anymore… I didn't think anything of it, of course—you see those patterns—but I saw 2 or 3 frogs at a spot one year and then went back 5 years later and didn't see anything. It didn't mean anything [at the time], but when you don't see them any more times that you're out there…then you realize that they're gone. (Interview 2)

Historical information can help guard against inaccurate assumptions about abundance and trends that can lead to poorly informed management decisions. Considering the extent of *R*. *boylii*'s extirpation from many areas of California, biologists are examining the practicalities of reintroducing the species to areas where it has been extirpated (Lind, 2005). Gaining a better understanding of pattern and process of extinctions and extirpations is only the beginning of successfully predicting best management practices for repatriation programs. An important next phase is to describe the immunogenetic and ecological attributes of *R*. *boylii* populations that are persisting despite ongoing Bd infection, and use insights gained from these studies to design *R*. *boylii* reintroduction programs that maximize the probability of success. In combination with our findings about the current prevalence of Bd among the amphibians dwelling in the streams that could provide suitable habitat for *R*. *boylii*, our historical analysis provides a solid foundation for assessing the feasibility of repatriation of this species to southern California where it has long been extirpated.

## CONFLICT OF INTEREST

None declared.

## AUTHORSHIP

AJA designed the study, acquired the data, analyzed and interpreted the data, and wrote the manuscript; APP contributed to acquisition and interpretation of data and revised the manuscript; CJB contributed to conception and design and analysis and interpretation of data and revised the manuscript.
