# Supplementary material for: Rapid extirpation of a North American frog coincides with an increase in fungal pathogen prevalence: Historical analysis and implications for reintroduction
Source: Ecol Evol. 2017 Oct 25;7(23):10216–32. doi: 10.1002/ece3.3468 (PMC5723621; doi:10.1002/ece3.3468)
Supplement: Supplementary file 1 [file ECE3-7-10216-s001.pdf]

Appendix 1

Questions asked during semi-directive interviews.

- Where did you grow up?
- How did you become interested in herps?
- What is your experience with herps in California or the American Southwest?
- How many years' experience do you have herping or conducting natural history observations in southern California?
- What is your age?
- Have you ever witnessed a local or regional amphibian die-off or mortality event?
- Have you ever witnessed a local or regional die-off of other (non-amphibian) taxa during your time in the field?
- Do you recall perceiving a pattern of amphibian declines prior to 1980? Prior to 1990?
- What factor(s) do you perceive as primary cause(s) of amphibian declines in California? Elsewhere?
- When was the last time you saw *Rana boylii* in southern California? In central California?
- In what relative abundance did you see *Rana boylii* at different times and in different localities?
- Do you have any un-archived field notes available from your *Rana boylii* observations? Of other species when you observed die-offs or declines?
- When did you first observe bullfrogs in southern California, and where?
- Have you seen *Xenopus laevis* in the wild in southern California?
