# Supplementary material for: Rapid extirpation of a North American frog coincides with an increase in fungal pathogen prevalence: Historical analysis and implications for reintroduction
Source: Ecol Evol. 2017 Oct 25;7(23):10216–32. doi: 10.1002/ece3.3468 (PMC5723621; doi:10.1002/ece3.3468)
Supplement: Supplementary file 2 [file ECE3-7-10216-s002.pdf]

Appendix 2. Museum specimen data used for analysis. Reps positive = number of qPCR replicates (out of 3) in which Bd was detected. Species codes are available in Figure 3 of the main text.

Institution	Catalog #	Species	County	Year Collected	Reps Positive
UCSB	6721	RADR	Santa Barbara	1975	0
UCSB	8023	RADR	Ventura	1979	0
UCSB	9427	HYRE	Santa Barbara	1973	1
UCSB	9836	HYCA	Santa Barbara	1979	0
UCSB	9837	HYCA	Santa Barbara	1979	0
UCSB	9838	HYCA	Santa Barbara	1979	0
UCSB	9839	HYCA	Santa Barbara	1979	0
UCSB	9840	HYCA	Santa Barbara	1979	0
UCSB	9841	HYCA	Ventura	1979	0
UCSB	9842	HYCA	Ventura	1979	0
UCSB	9843	HYRE	Santa Barbara	1979	0
UCSB	9852	RADR	Santa Barbara	1979	2
UCSB	9853	RADR	Santa Barbara	1979	2
UCSB	9854	RADR	Santa Barbara	1979	1
UCSB	9855	RADR	Santa Barbara	1979	0
UCSB	9966	HYRE	Ventura	1979	2
UCSB	9967	HYRE	Ventura	1979	3
UCSB	9968	HYRE	Ventura	1979	3
UCSB	9969	HYRE	Ventura	1979	3
UCSB	9970	HYRE	Ventura	1979	3
UCSB	9972	HYCA	Santa Barbara	1979	0
UCSB	9973	HYCA	Santa Barbara	1979	0
UCSB	9974	HYCA	Santa Barbara	1979	0
UCSB	9975	HYCA	Santa Barbara	1979	0
UCSB	9977	HYCA	Santa Barbara	1979	0
UCSB	9978	HYCA	Santa Barbara	1979	0
UCSB	9979	HYCA	Santa Barbara	1979	0
UCSB	9980	HYCA	Santa Barbara	1979	0
UCSB	9981	HYCA	Santa Barbara	1979	0
UCSB	9982	HYCA	Santa Barbara	1979	0
UCSB	9983	HYCA	Santa Barbara	1979	0
UCSB	9984	HYCA	Santa Barbara	1979	0
UCSB	9985	HYCA	Santa Barbara	1979	0
UCSB	10010	HYRE	Santa Barbara	1979	0
UCSB	10011	HYCA	Santa Barbara	1979	0
UCSB	10012	HYCA	Santa Barbara	1979	0
UCSB	10013	HYCA	Santa Barbara	1979	0
UCSB	10014	HYCA	Santa Barbara	1979	0
UCSB	10015	HYCA	Santa Barbara	1979	0
UCSB	10016	HYCA	Santa Barbara	1979	0
UCSB	10017	HYCA	Santa Barbara	1979	0

Appendix 2. Museum specimen data used for analysis. Reps positive = number of qPCR replicates (out of 3) in which Bd was detected. Species codes are available in Figure 3 of the main text.

Institution	Catalog #	Species	County	Year Collected	Reps Positive
UCSB	10018	HYCA	Santa Barbara	1979	0
UCSB	10019	HYCA	Santa Barbara	1979	0
UCSB	10020	HYCA	Santa Barbara	1979	0
UCSB	10021	HYCA	Santa Barbara	1979	0
UCSB	10022	HYCA	Santa Barbara	1979	0
UCSB	10023	HYCA	Santa Barbara	1979	0
UCSB	10024	HYCA	Santa Barbara	1979	0
UCSB	10025	HYCA	Santa Barbara	1979	0
UCSB	10026	HYCA	Santa Barbara	1979	0
UCSB	10027	HYCA	Ventura	1979	0
UCSB	10028	HYCA	Ventura	1979	0
UCSB	10029	HYCA	Ventura	1979	0
UCSB	10030	HYCA	Ventura	1979	0
UCSB	10031	HYCA	Ventura	1979	0
UCSB	10032	HYCA	Ventura	1979	0
UCSB	10033	HYCA	Ventura	1979	0
UCSB	10034	HYCA	Ventura	1979	1
UCSB	10035	HYCA	Ventura	1979	0
UCSB	10036	HYCA	Ventura	1979	0
UCSB	10037	HYCA	Ventura	1979	0
UCSB	10038	HYCA	Ventura	1979	3
UCSB	10039	HYCA	Ventura	1980	0
UCSB	10040	HYCA	Ventura	1980	0
UCSB	10041	HYCA	Ventura	1980	0
UCSB	10042	HYCA	Ventura	1980	0
UCSB	10043	HYCA	Ventura	1980	3
UCSB	10044	HYCA	Ventura	1980	3
UCSB	10045	HYCA	Ventura	1980	3
UCSB	10046	HYCA	Ventura	1980	1
UCSB	10047	HYCA	Ventura	1980	0
UCSB	10048	HYCA	Ventura	1980	1
UCSB	10061	HYCA	Ventura	1979	0
UCSB	10062	HYCA	Ventura	1979	0
UCSB	10063	HYCA	Ventura	1979	1
UCSB	10064	HYRE	Ventura	1979	1
UCSB	10065	HYRE	Ventura	1979	1
UCSB	10066	HYRE	Ventura	1979	0
UCSB	10079	HYCA	Santa Barbara	1979	1
UCSB	10081	HYRE	Santa Barbara	1980	0
UCSB	10089	HYCA	Santa Barbara	1980	0
UCSB	11301	RADR	Santa Barbara	1980	0

Appendix 2. Museum specimen data used for analysis. Reps positive = number of qPCR replicates (out of 3) in which Bd was detected. Species codes are available in Figure 3 of the main text.

Institution	Catalog #	Species	County	Year Collected	Reps Positive
UCSB	11371	RADR	Santa Barbara	1979	0
UCSB	11398	ANBO	Santa Barbara	1980	0
UCSB	11461	HYRE	Santa Barbara	1981	3
UCSB	11516	HYCA	Santa Barbara	1980	0
UCSB	11517	HYCA	Santa Barbara	1980	0
UCSB	11518	HYCA	Santa Barbara	1980	0
UCSB	11519	HYCA	Santa Barbara	1980	0
UCSB	11520	HYRE	Santa Barbara	1979	0
UCSB	11521	HYRE	Santa Barbara	1979	0
UCSB	11522	HYCA	Santa Barbara	1979	0
UCSB	11523	HYCA	Santa Barbara	1979	0
UCSB	11524	HYCA	Santa Barbara	1979	0
UCSB	11525	HYCA	Santa Barbara	1979	0
UCSB	11526	HYCA	Ventura	1980	0
UCSB	11527	HYCA	Ventura	1980	0
UCSB	11528	HYCA	Ventura	1980	1
UCSB	11529	HYCA	Ventura	1980	0
UCSB	11539	HYCA	Ventura	1980	0
UCSB	11540	HYCA	Ventura	1980	0
UCSB	11615	HYRE	Los Angeles	1980	0
UCSB	11616	HYRE	Los Angeles	1980	0
UCSB	11617	HYRE	Los Angeles	1980	0
UCSB	11618	HYRE	Los Angeles	1980	1
UCSB	11619	HYRE	Los Angeles	1980	0
UCSB	11620	HYRE	Los Angeles	1980	0
UCSB	11623	HYRE	Los Angeles	1980	3
UCSB	11624	HYRE	Los Angeles	1980	2
UCSB	11625	HYRE	Los Angeles	1980	3
UCSB	11626	HYRE	Los Angeles	1980	2
UCSB	11627	HYRE	Los Angeles	1980	3
UCSB	11628	HYRE	Los Angeles	1980	3
UCSB	11629	HYRE	Los Angeles	1980	0
UCSB	11630	HYRE	Los Angeles	1980	0
UCSB	11631	HYRE	Los Angeles	1980	0
UCSB	11632	HYRE	Los Angeles	1980	1
UCSB	11633	HYRE	Los Angeles	1980	2
UCSB	11634	HYRE	Los Angeles	1980	2
UCSB	11635	HYRE	Los Angeles	1980	2
UCSB	11638	HYCA	Ventura	1981	3
UCSB	11639	HYCA	Ventura	1981	2
UCSB	11641	HYCA	Ventura	1981	1

Appendix 2. Museum specimen data used for analysis. Reps positive = number of qPCR replicates (out of 3) in which Bd was detected. Species codes are available in Figure 3 of the main text.

Institution	Catalog #	Species	County	Year Collected	Reps Positive
UCSB	11642	HYCA	Ventura	1981	0
UCSB	11643	HYCA	Ventura	1981	0
UCSB	11644	HYCA	Ventura	1981	0
UCSB	11645	HYCA	Ventura	1981	0
UCSB	11646	HYCA	Ventura	1981	0
UCSB	11647	HYCA	Santa Barbara	1979	0
UCSB	11648	HYCA	Santa Barbara	1979	0
UCSB	11649	HYCA	Santa Barbara	1979	0
UCSB	11650	HYCA	Santa Barbara	1979	0
UCSB	11651	HYCA	Santa Barbara	1979	0
UCSB	11652	HYCA	Santa Barbara	1979	0
UCSB	11653	HYCA	Santa Barbara	1979	0
UCSB	11654	HYCA	Santa Barbara	1979	0
UCSB	11655	HYCA	Santa Barbara	1979	0
UCSB	11656	HYCA	Santa Barbara	1979	0
UCSB	11657	HYCA	Santa Barbara	1979	0
UCSB	11681	HYRE	Ventura	1979	0
UCSB	11682	HYRE	Ventura	1979	0
UCSB	11683	HYRE	Ventura	1979	0
UCSB	11684	HYRE	Ventura	1979	0
UCSB	11685	HYRE	Ventura	1979	0
UCSB	11686	HYRE	Ventura	1979	1
UCSB	11687	HYRE	Ventura	1979	1
UCSB	11688	HYRE	Ventura	1979	0
UCSB	11689	RACA	Ventura	1979	0
UCSB	11700	HYRE	Santa Barbara	1981	0
UCSB	11701	HYRE	Santa Barbara	1981	3
UCSB	11705	HYCA	Ventura	1981	1
UCSB	11706	HYCA	Ventura	1981	2
UCSB	11707	HYRE	Santa Barbara	1981	0
UCSB	11708	HYRE	Ventura	1981	0
UCSB	11745	RADR	Santa Barbara	1980	2
UCSB	11747	HYRE	Santa Barbara	1980	0
UCSB	11816	HYRE	Ventura	1979	0
UCSB	11818	HYRE	Santa Barbara	1979	1
UCSB	11819	HYCA	Ventura	1979	1
UCSB	11820	HYCA	Santa Barbara	1979	0
UCSB	11821	HYRE	Ventura	1981	0
UCSB	11889	HYRE	Santa Barbara	1980	0
UCSB	11891	HYCA	Santa Barbara	1979	0
UCSB	11892	HYCA	Santa Barbara	1979	1

Appendix 2. Museum specimen data used for analysis. Reps positive = number of qPCR replicates (out of 3) in which Bd was detected. Species codes are available in Figure 3 of the main text.

Institution	Catalog #	Species	County	Year Collected	Reps Positive
UCSB	11893	HYCA	Santa Barbara	1979	0
UCSB	11894	HYCA	Santa Barbara	1979	0
UCSB	11895	HYCA	Santa Barbara	1979	0
UCSB	11896	HYCA	Santa Barbara	1979	0
UCSB	11897	HYCA	Santa Barbara	1979	0
UCSB	11898	HYCA	Santa Barbara	1979	0
UCSB	11899	HYCA	Santa Barbara	1979	0
UCSB	11900	HYCA	Santa Barbara	1979	0
UCSB	11901	HYCA	Santa Barbara	1979	0
UCSB	11902	HYCA	Santa Barbara	1979	0
UCSB	11903	HYCA	Santa Barbara	1979	0
UCSB	11904	HYCA	Santa Barbara	1979	0
UCSB	11905	HYCA	Santa Barbara	1979	1
UCSB	11906	HYCA	Santa Barbara	1979	0
UCSB	11907	HYCA	Santa Barbara	1979	0
UCSB	11908	HYCA	Santa Barbara	1979	0
UCSB	11909	HYCA	Santa Barbara	1979	0
UCSB	11910	HYCA	Santa Barbara	1979	0
UCSB	11911	HYCA	Santa Barbara	1979	0
UCSB	11912	HYCA	Santa Barbara	1979	0
UCSB	11913	HYCA	Santa Barbara	1979	0
UCSB	11914	HYCA	Santa Barbara	1979	0
UCSB	11915	HYRE	Santa Barbara	1979	0
UCSB	11923	HYCA	Ventura	1979	0
UCSB	11924	HYCA	Ventura	1979	0
UCSB	11925	HYCA	Ventura	1979	0
UCSB	11926	HYCA	Ventura	1979	0
UCSB	11927	HYCA	Ventura	1979	0
UCSB	11928	HYCA	Ventura	1979	0
UCSB	11929	HYCA	Ventura	1979	0
UCSB	11930	HYCA	Ventura	1979	0
UCSB	11932	HYCA	Ventura	1979	0
UCSB	11933	HYCA	Ventura	1979	0
UCSB	11934	HYCA	Ventura	1979	3
UCSB	11937	HYCA	Ventura	1979	0
UCSB	11939	HYCA	Ventura	1979	0
UCSB	11940	HYCA	Ventura	1979	0
UCSB	11941	HYCA	Ventura	1979	0
UCSB	11943	HYCA	Ventura	1979	0
UCSB	11944	HYCA	Ventura	1979	0
UCSB	11946	HYCA	Ventura	1979	0

Appendix 2. Museum specimen data used for analysis. Reps positive = number of qPCR replicates (out of 3) in which Bd was detected. Species codes are available in Figure 3 of the main text.

Institution	Catalog #	Species	County	Year Collected	Reps Positive
UCSB	11947	HYCA	Ventura	1979	0
UCSB	11950	HYCA	Ventura	1979	0
UCSB	11951	HYCA	Ventura	1979	0
UCSB	12278	RADR	Santa Barbara	1981	0
UCSB	12535	HYRE	Los Angeles	1981	1
UCSB	12536	HYRE	Los Angeles	1981	1
UCSB	12600	HYRE	Ventura	1980	0
UCSB	12601	HYRE	Ventura	1980	0
UCSB	12607	HYRE	Ventura	1980	0
UCSB	12608	HYRE	Ventura	1980	0
UCSB	12609	HYCA	Ventura	1980	0
UCSB	12610	HYRE	Ventura	1980	0
UCSB	12612	HYRE	Ventura	1981	3
UCSB	12613	HYRE	Ventura	1981	0
UCSB	12615	HYRE	Ventura	1981	3
UCSB	12641	HYCA	Santa Barbara	1979	0
UCSB	12642	HYCA	Santa Barbara	1979	0
UCSB	12647	HYCA	Ventura	1979	0
UCSB	12648	HYCA	Ventura	1979	0
UCSB	12666	HYCA	Ventura	1981	0
UCSB	12667	HYCA	Ventura	1981	0
UCSB	12668	HYCA	Ventura	1981	0
UCSB	12669	HYCA	Ventura	1981	0
UCSB	12670	HYCA	Ventura	1981	0
UCSB	12671	HYRE	Santa Barbara	1981	0
UCSB	12672	HYCA	Ventura	1981	1
UCSB	12673	HYCA	Ventura	1981	0
UCSB	12674	HYCA	Ventura	1981	0
UCSB	12675	HYCA	Santa Barbara	1980	0
UCSB	12676	HYCA	Santa Barbara	1980	0
UCSB	12677	HYCA	Santa Barbara	1980	0
UCSB	12678	HYCA	Santa Barbara	1980	0
UCSB	12681	HYCA	Santa Barbara	1980	0
UCSB	12682	HYCA	Santa Barbara	1979	0
UCSB	12683	HYCA	Ventura	1980	0
UCSB	12684	HYCA	Ventura	1980	0
UCSB	12685	HYCA	Ventura	1980	1
UCSB	12686	HYCA	Ventura	1980	0
UCSB	12692	HYRE	Santa Barbara	1980	0
UCSB	12769	HYRE	Ventura	1981	0
UCSB	12770	HYRE	Ventura	1981	0

Appendix 2. Museum specimen data used for analysis. Reps positive = number of qPCR replicates (out of 3) in which Bd was detected. Species codes are available in Figure 3 of the main text.

Institution	Catalog #	Species	County	Year Collected	Reps Positive
UCSB	12771	HYRE	Ventura	1981	0
UCSB	12772	HYRE	Ventura	1981	0
UCSB	13042	RADR	Ventura	1981	1
UCSB	13578	HYCA	Ventura	1982	0
UCSB	13579	HYCA	Ventura	1982	3
UCSB	13580	HYCA	Ventura	1982	0
UCSB	13581	HYCA	Ventura	1982	3
UCSB	13582	HYCA	Ventura	1982	0
UCSB	13583	HYCA	Santa Barbara	1982	1
UCSB	13800	HYRE	Santa Barbara	1979	2
UCSB	13936	HYRE	Los Angeles	1982	3
UCSB	13937	HYRE	Los Angeles	1982	3
UCSB	13938	HYRE	Los Angeles	1982	3
UCSB	14134	HYRE	Santa Barbara	1982	1
UCSB	14137	HYRE	Santa Barbara	1982	0
UCSB	14138	HYRE	Los Angeles	1982	2
UCSB	14552	RADR	Santa Barbara	1982	2
UCSB	14573	HYCA	Ventura	1981	0
UCSB	14574	HYCA	Ventura	1981	0
UCSB	14575	HYCA	Ventura	1981	0
UCSB	14576	HYCA	Ventura	1981	0
UCSB	14577	HYCA	Santa Barbara	1982	0
UCSB	14578	HYCA	Santa Barbara	1982	0
UCSB	14586	HYRE	Santa Barbara	1982	0
UCSB	14824	HYRE	Santa Barbara	1982	0
UCSB	14884	RADR	Santa Barbara	1982	1
UCSB	15019	HYRE	Ventura	1983	2
UCSB	15020	HYRE	Ventura	1983	0
UCSB	15021	HYRE	Ventura	1983	2
UCSB	15022	HYRE	Ventura	1983	0
UCSB	15285	RACA	Ventura	1983	0
UCSB	15286	RACA	Ventura	1983	0
UCSB	15287	RACA	Ventura	1983	3
UCSB	15288	RACA	Ventura	1983	0
UCSB	15289	RACA	Ventura	1983	3
UCSB	15290	RACA	Ventura	1983	0
UCSB	15291	RACA	Ventura	1983	0
UCSB	15292	RACA	Ventura	1983	1
UCSB	15294	RACA	Ventura	1983	1
UCSB	15891	HYCA	Santa Barbara	1984	0
UCSB	15892	HYCA	Santa Barbara	1984	0

Appendix 2. Museum specimen data used for analysis. Reps positive = number of qPCR replicates (out of 3) in which Bd was detected. Species codes are available in Figure 3 of the main text.

Institution	Catalog #	Species	County	Year Collected	Reps Positive
UCSB	15893	HYCA	Santa Barbara	1984	0
UCSB	15894	HYCA	Santa Barbara	1984	0
UCSB	15895	HYCA	Santa Barbara	1984	1
UCSB	16891	RADR	Santa Barbara	1984	0
UCSB	16892	RADR	Santa Barbara	1984	2
UCSB	17132	RADR	Santa Barbara	1984	0
UCSB	17170	HYCA	Ventura	1984	0
UCSB	17171	HYCA	Ventura	1984	0
UCSB	17172	HYCA	Ventura	1984	0
UCSB	17173	HYCA	Ventura	1984	0
UCSB	17174	HYCA	Ventura	1984	0
UCSB	17175	HYCA	Santa Barbara	1984	0
UCSB	17608	HYCA	Ventura	1981	0
UCSB	17632	HYCA	Ventura	1984	0
UCSB	17836	HYRE	Los Angeles	1985	0
UCSB	19547	HYCA	Ventura	1982	0
UCSB	22305	HYCA	Ventura	1988	2
UCSB	22306	HYCA	Ventura	1988	3
UCSB	23667	HYCA	Ventura	1989	3
UCSB	23668	HYCA	Ventura	1989	0
UCSB	24674	HYCA	Ventura	1988	0
UCSB	24948	RACA	Ventura	1990	0
UCSB	24949	RACA	Ventura	1990	0
UCSB	24950	RACA	Ventura	1990	0
UCSB	25120	RADR	Santa Barbara	1990	3
UCSB	25148	RACA	Ventura	1990	0
UCSB	26543	HYRE	Ventura	1991	1
UCSB	26544	HYRE	Ventura	1991	0
UCSB	27716	RADR	Santa Barbara	1993	3
UCSB	27843	RADR	Santa Barbara	1993	3
UCSB	28310	RADR	Santa Barbara	1995	3
UCSB	28422	RADR	Santa Barbara	1995	3
UCSB	28450	RADR	Santa Barbara	1995	3
UCSB	28985	RADR	Santa Barbara	1995	3
UCSB	28995	RADR	Santa Barbara	1995	3
UCSB	28996	RADR	Santa Barbara	1995	3
UCSB	28997	RADR	Santa Barbara	1995	3
UCSB	28998	RADR	Santa Barbara	1995	2
UCSB	28999	RADR	Santa Barbara	1995	0
UCSB	29284	RADR	Santa Barbara	1995	0
UCSB	29288	RADR	Santa Barbara	1995	3

Appendix 2. Museum specimen data used for analysis. Reps positive = number of qPCR replicates (out of 3) in which Bd was detected. Species codes are available in Figure 3 of the main text.

Institution	Catalog #	Species	County	Year Collected	Reps Positive
UCSB	29290	RADR	Santa Barbara	1995	3
UCSB	29291	RADR	Santa Barbara	1995	2
UCSB	29309	RACA	Santa Barbara	1996	0
UCSB	29310	RACA	Santa Barbara	1996	0
UCSB	29312	RACA	Santa Barbara	1996	0
UCSB	29314	RACA	Santa Barbara	1996	0
UCSB	29315	RACA	Santa Barbara	1996	0
UCSB	29316	RACA	Santa Barbara	1996	3
UCSB	29317	RACA	Santa Barbara	1996	0
UCSB	29318	RACA	Santa Barbara	1996	2
UCSB	29319	RACA	Santa Barbara	1996	0
UCSB	29320	RACA	Santa Barbara	1996	0
UCSB	29321	RACA	Santa Barbara	1996	3
UCSB	29322	RACA	Santa Barbara	1996	1
UCSB	29324	RACA	Santa Barbara	1996	0
UCSB	29328	RACA	Santa Barbara	1996	2
UCSB	29331	RACA	Santa Barbara	1996	1
UCSB	29332	RACA	Santa Barbara	1996	0
UCSB	29342	RACA	Santa Barbara	1996	0
UCSB	29343	RACA	Santa Barbara	1996	1
UCSB	29344	RACA	Santa Barbara	1996	0
UCSB	29361	RADR	Santa Barbara	1990	0
UCSB	29380	HYCA	Ventura	1990	0
UCSB	29436	RADR	Santa Barbara	1995	3
UCSB	29827	RADR	San Luis Obispo	1995	0
UCSB	29828	RADR	San Luis Obispo	1995	0
UCSB	29829	RADR	San Luis Obispo	1995	1
UCSB	29831	RADR	San Luis Obispo	1995	0
UCSB	29832	RADR	San Luis Obispo	1995	0
UCSB	29833	RADR	San Luis Obispo	1995	1
UCSB	30136	RACA	Santa Barbara	2004	3
UCSB	30525	RADR	San Luis Obispo	1995	1
UCSB	30637	RADR	Santa Barbara	2009	0
UCSB	31101	RADR	Santa Barbara	1995	3
SDNHM	2316	TATO	Los Angeles	1930	0
SDNHM	13295	TATO	Los Angeles	1930	0
SDNHM	13296	TATO	Los Angeles	1930	0
SDNHM	14276	TATO	Los Angeles	1930	0
SDNHM	14277	TATO	Los Angeles	1930	0
SDNHM	14282	TATO	Los Angeles	1930	0
SDNHM	14283	TATO	Los Angeles	1930	0

Appendix 2. Museum specimen data used for analysis. Reps positive = number of qPCR replicates (out of 3) in which Bd was detected. Species codes are available in Figure 3 of the main text.

Institution	Catalog #	Species	County	Year Collected	Reps Positive
SDNHM	14286	TATO	Los Angeles	1930	0
SDNHM	14288	TATO	Los Angeles	1930	0
SDNHM	14292	TATO	Los Angeles	1930	0
SDNHM	14294	TATO	Los Angeles	1930	0
SDNHM	14296	TATO	Los Angeles	1930	0
SDNHM	14297	TATO	Los Angeles	1930	0
SDNHM	19525	TATO	Los Angeles	1958	0
SDNHM	19526	TATO	Los Angeles	1958	0
SDNHM	19527	TATO	Los Angeles	1958	0
SDNHM	19528	TATO	Los Angeles	1958	0
SDNHM	19529	TATO	Los Angeles	1958	0
SDNHM	19530	TATO	Los Angeles	1958	0
SDNHM	19532	TATO	Los Angeles	1958	0
SDNHM	41736	TATO	Los Angeles	1948	0
SDNHM	41740	TATO	Los Angeles	1948	0
SDNHM	41742	TATO	Los Angeles	1948	0
SDNHM	41743	TATO	Los Angeles	1948	0
SDNHM	41744	TATO	Los Angeles	1948	0
SDNHM	41745	TATO	Los Angeles	1948	0
SDNHM	41746	TATO	Los Angeles	1948	0
SDNHM	41747	TATO	Los Angeles	1948	0
SDNHM	41748	TATO	Los Angeles	1948	0
SDNHM	13272	ANBO	Los Angeles	1930	0
SDNHM	13275	ANBO	Los Angeles	1930	0
SDNHM	13276	ANBO	Los Angeles	1930	0
SDNHM	13277	HYRE	Los Angeles	1930	0
SDNHM	13278	HYRE	Los Angeles	1930	0
SDNHM	13280	HYRE	Los Angeles	1930	0
SDNHM	13282	HYRE	Los Angeles	1930	0
SDNHM	13285	HYRE	Los Angeles	1930	0
SDNHM	13286	HYRE	Los Angeles	1930	0
SDNHM	41749	TATO	Los Angeles	1948	0
SDNHM	47766	TATO	Los Angeles	1962	0
SDNHM	47767	TATO	Los Angeles	1962	0
SDNHM	47768	TATO	Los Angeles	1962	3
SDNHM	19252	HYCA	Los Angeles	1958	0
SDNHM	19253	HYCA	Los Angeles	1958	0
SDNHM	19255	HYCA	Los Angeles	1958	0
SDNHM	19256	HYCA	Los Angeles	1958	0
SDNHM	19259	HYCA	Los Angeles	1958	0
SDNHM	19261	HYCA	Los Angeles	1958	0

Appendix 2. Museum specimen data used for analysis. Reps positive = number of qPCR replicates (out of 3) in which Bd was detected. Species codes are available in Figure 3 of the main text.

Institution	Catalog #	Species	County	Year Collected	Reps Positive
SDNHM	19262	HYCA	Los Angeles	1958	0
SDNHM	19263	HYCA	Los Angeles	1958	0
SDNHM	19534	RADR	Los Angeles	1958	0
SDNHM	19535	RADR	Los Angeles	1958	0
SDNHM	19536	RADR	Los Angeles	1958	0
SDNHM	19537	RADR	Los Angeles	1958	0
SDNHM	19538	RADR	Los Angeles	1958	0
SDNHM	19540	RADR	Los Angeles	1958	0
SDNHM	19541	RADR	Los Angeles	1958	0
SDNHM	19542	RADR	Los Angeles	1958	0
SDNHM	19543	RADR	Los Angeles	1958	0
SDNHM	19544	RADR	Los Angeles	1958	0
SDNHM	19545	RADR	Los Angeles	1958	0
SDNHM	20774	RADR	Santa Barbara	1933	0
SDNHM	20775	RADR	Santa Barbara	1933	0
SDNHM	20777	HYRE	Santa Barbara	1933	1
SDNHM	20778	HYRE	Santa Barbara	1933	0
SDNHM	33459	RADR	Santa Barbara	1940	0
SDNHM	33460	ANBO	Santa Barbara	1940	0
SDNHM	33780	HYCA	Santa Barbara	1941	0
SDNHM	58376	TATO	San Luis Obispo	1974	0
SDNHM	59421	TATO	San Luis Obispo	1977	0
SDNHM	47510	HYRE	Santa Barbara	1968	0
SDNHM	47511	HYRE	Santa Barbara	1968	0
SDNHM	53677	HYRE	San Luis Obispo	1962	0
SDNHM	54344	HYRE	Los Angeles	1962	0
SDNHM	54345	HYRE	Los Angeles	1962	0
SDNHM	54346	HYRE	Los Angeles	1962	0
SDNHM	54347	HYRE	Los Angeles	1962	0
SDNHM	54348	HYRE	Los Angeles	1962	0
SDNHM	54349	HYRE	Los Angeles	1962	0
SDNHM	54350	HYRE	Los Angeles	1962	0
SDNHM	54351	HYRE	Los Angeles	1962	0
SDNHM	54352	HYRE	Los Angeles	1962	0
SDNHM	54353	HYRE	Los Angeles	1962	0
SDNHM	54354	HYRE	Los Angeles	1962	1
SDNHM	54355	HYRE	Los Angeles	1962	0
SDNHM	54356	HYRE	Los Angeles	1962	0
SDNHM	54357	HYRE	Los Angeles	1962	0
SDNHM	54358	HYRE	Los Angeles	1962	0
SDNHM	54359	HYRE	Los Angeles	1962	0

Appendix 2. Museum specimen data used for analysis. Reps positive = number of qPCR replicates (out of 3) in which Bd was detected. Species codes are available in Figure 3 of the main text.

Institution	Catalog #	Species	County	Year Collected	Reps Positive
SDNHM	54360	HYRE	Los Angeles	1962	0
SDNHM	54361	HYRE	Los Angeles	1962	0
SDNHM	54362	HYRE	Los Angeles	1962	0
SDNHM	54363	HYRE	Los Angeles	1962	0
SDNHM	54364	HYRE	Los Angeles	1962	0
SDNHM	54365	HYRE	Los Angeles	1962	0
SDNHM	54366	HYRE	Los Angeles	1962	0
SDNHM	54368	HYRE	Los Angeles	1962	0
SDNHM	54370	HYRE	Los Angeles	1962	0
SDNHM	54371	HYRE	Los Angeles	1962	0
SDNHM	55350	HYCA	Los Angeles	1974	0
SDNHM	56697	ANBO	Los Angeles	1975	0
SDNHM	56700	ANBO	Los Angeles	1975	0
SDNHM	56704	ANBO	Los Angeles	1975	0
SDNHM	56705	ANBO	Los Angeles	1975	0
SDNHM	56706	ANBO	Los Angeles	1975	0
SDNHM	56707	ANBO	Los Angeles	1975	0
SDNHM	56708	ANBO	Los Angeles	1975	0
SDNHM	56709	ANBO	Los Angeles	1975	0
SDNHM	56710	ANBO	Los Angeles	1975	0
SDNHM	56711	ANBO	Los Angeles	1975	0
SDNHM	57026	HYRE	Los Angeles	1971	0
SDNHM	57027	HYRE	Los Angeles	1971	0
SDNHM	58269	HYRE	Santa Barbara	1975	3
SDNHM	58273	HYRE	Santa Barbara	1975	3
SDNHM	58274	HYRE	Santa Barbara	1975	1
SDNHM	58276	HYRE	Santa Barbara	1975	3
SDNHM	58277	HYRE	Santa Barbara	1975	2
SDNHM	58278	HYRE	Santa Barbara	1975	0
SDNHM	58279	RADR	Santa Barbara	1974	3
SDNHM	58283	ANBO	Santa Barbara	1974	0
SDNHM	58284	ANBO	Santa Barbara	1975	0
SDNHM	58285	ANBO	Santa Barbara	1975	0
SDNHM	58375	RADR	Santa Barbara	1974	0
SDNHM	75752	HYCA	Los Angeles	2009	0
SDNHM	75753	HYCA	Los Angeles	2009	0
SDNHM	75754	HYCA	Los Angeles	2009	2
SDNHM	34357	TATO	Santa Barbara	1941	0
SDNHM	34358	TATO	Santa Barbara	1941	1
SDNHM	34359	TATO	Santa Barbara	1941	0
SDNHM	20776	RABO	Santa Barbara	1933	0

Appendix 2. Museum specimen data used for analysis. Reps positive = number of qPCR replicates (out of 3) in which Bd was detected. Species codes are available in Figure 3 of the main text.

Institution	Catalog #	Species	County	Year Collected	Reps Positive
SBMNH	85	HYRE	Santa Barbara	1963	0
SBMNH	86	HYRE	Santa Barbara	1963	0
SBMNH	87	HYRE	Santa Barbara	1963	0
SBMNH	88	HYCA	Santa Barbara	1963	0
SBMNH	89	HYRE	Santa Barbara	1963	0
SBMNH	90	HYRE	Santa Barbara	1963	0
SBMNH	91	HYRE	Santa Barbara	1963	0
SBMNH	92	HYRE	Santa Barbara	1963	0
SBMNH	93	HYRE	Santa Barbara	1963	0
SBMNH	94	HYRE	Santa Barbara	1963	0
SBMNH	95	HYRE	Santa Barbara	1963	0
SBMNH	96	HYRE	Santa Barbara	1963	0
SBMNH	98	HYCA	Santa Barbara	1963	0
SBMNH	99	HYRE	Santa Barbara	1963	0
SBMNH	106	RADR	Santa Barbara	1963	0
SBMNH	107	RADR	Santa Barbara	1963	0
SBMNH	108	RADR	Santa Barbara	1963	0
SBMNH	109	RADR	Santa Barbara	1963	0
SBMNH	110	RADR	Santa Barbara	1963	0
SBMNH	111	RADR	Santa Barbara	1963	0
SBMNH	112	HYCA	Santa Barbara	1963	0
SBMNH	113	HYCA	Santa Barbara	1963	0
SBMNH	114	HYRE	Santa Barbara	1963	0
SBMNH	115	HYCA	Santa Barbara	1963	0
SBMNH	116	HYCA	Santa Barbara	1963	0
SBMNH	117	HYCA	Santa Barbara	1963	0
SBMNH	118	HYCA	Santa Barbara	1963	0
SBMNH	119	HYCA	Santa Barbara	1963	0
SBMNH	120	HYCA	Santa Barbara	1963	0
SBMNH	121	HYCA	Santa Barbara	1963	0
SBMNH	122	HYCA	Santa Barbara	1963	0
SBMNH	264	TATO	Los Angeles	1953	0
SBMNH	142	RADR	San Luis Obispo	1963	0
SBMNH	187	HYRE	Santa Barbara	1982	0
SBMNH	265	TATO	Los Angeles	1953	0
SBMNH	221	ANBO	Santa Barbara	1974	0
SBMNH	222	HYRE	Santa Barbara	1974	3
SBMNH	226	RADR	Santa Barbara	1974	3
SBMNH	280	HYRE	Santa Barbara	1974	3
SBMNH	281	HYCA	Santa Barbara	1974	3
SBMNH	284	RADR	San Luis Obispo	1963	1

Appendix 2. Museum specimen data used for analysis. Reps positive = number of qPCR replicates (out of 3) in which Bd was detected. Species codes are available in Figure 3 of the main text.

Institution	Catalog #	Species	County	Year Collected	Reps Positive
SBMNH	316	RADR	Santa Barbara	1975	1
SBMNH	317	RADR	Santa Barbara	1975	0
SBMNH	390	ANBO	Santa Barbara	1975	0
SBMNH	444	HYCA	Santa Barbara	1978	0
SBMNH	478	HYCA	Santa Barbara	1978	0
SBMNH	479	HYRE	Santa Barbara	1978	0
SBMNH	491	HYRE	Santa Barbara	1979	0
SBMNH	506	HYRE	Santa Barbara	1979	0
SBMNH	507	HYCA	Santa Barbara	1979	0
SBMNH	508	HYCA	Santa Barbara	1979	0
SBMNH	599	HYCA	Santa Barbara	1981	0
SBMNH	728	RACA	Santa Barbara	1981	0
SBMNH	743	ANBO	Santa Barbara	1982	0
SBMNH	1060	HYRE	Santa Barbara	1984	0
SBMNH	1067	ANBO	Santa Barbara	1984	0
SBMNH	1072	RADR	Santa Barbara	1984	0
SBMNH	1149	RADR	Santa Barbara	1985	0
SBMNH	1235	RACA	Santa Barbara	1986	0
SBMNH	1448	RACA	Santa Barbara	1989	0
SBMNH	1522	RADR	Santa Barbara	1991	0
SBMNH	2043	ANBO	Santa Barbara	2000	1
SBMNH	2158	RADR	Santa Barbara	2001	1
SBMNH	2159	RADR	Santa Barbara	2001	0
SBMNH	2160	RADR	Santa Barbara	2001	0
SBMNH	2162	RADR	Santa Barbara	2001	0
SBMNH	2166	RADR	Santa Barbara	2001	0
SBMNH	2186	HYRE	Santa Barbara	2001	0
SBMNH	2187	HYRE	Santa Barbara	2001	0
SBMNH	2188	HYRE	Santa Barbara	2001	0
SBMNH	2189	HYRE	Santa Barbara	2001	0
SBMNH	2217	RACA	Ventura	2001	0
SBMNH	2216	RACA	Ventura	2001	0
SBMNH	2382	HYRE	San Luis Obispo	2006	0
SBMNH	2387	HYRE	San Luis Obispo	2006	0
SBMNH	2388	HYRE	San Luis Obispo	2006	0
SBMNH	2389	HYRE	San Luis Obispo	2006	0
SBMNH	2390	HYRE	San Luis Obispo	2006	0
SBMNH	2450	HYRE	Los Angeles	2007	0
SBMNH	2451	HYRE	Los Angeles	2007	0
SBMNH	136	RABO	San Luis Obispo	1963	0
SBMNH	137	RABO	San Luis Obispo	1963	0

Appendix 2. Museum specimen data used for analysis. Reps positive = number of qPCR replicates (out of 3) in which Bd was detected. Species codes are available in Figure 3 of the main text.

Institution	Catalog #	Species	County	Year Collected	Reps Positive
SBMNH	138	RABO	San Luis Obispo	1963	0
SBMNH	139	RABO	San Luis Obispo	1963	0
SBMNH	140	RABO	San Luis Obispo	1963	0
SBMNH	135	TATO	San Luis Obispo	1963	0
SBMNH	215	TATO	Santa Barbara	1974	2
SBMNH	216	TATO	Santa Barbara	1974	2
SBMNH	217	TATO	Santa Barbara	1974	0
SBMNH	218	TATO	Santa Barbara	1974	3
SBMNH	568	TATO	Santa Barbara	1980	3
SBMNH	596	TATO	Santa Barbara	1980	3
SBMNH	620	TATO	Santa Barbara	1981	3
SBMNH	621	TATO	Santa Barbara	1981	3
SBMNH	622	TATO	Santa Barbara	1981	3
SBMNH	623	TATO	Santa Barbara	1981	3
SBMNH	624	TATO	Santa Barbara	1981	3
SBMNH	625	TATO	Santa Barbara	1981	3
SBMNH	626	TATO	Santa Barbara	1981	3
SBMNH	627	TATO	Santa Barbara	1981	3
SBMNH	628	TATO	Santa Barbara	1981	3
SBMNH	629	TATO	Santa Barbara	1981	3
SBMNH	1150	TATO	Santa Barbara	1985	0
SBMNH	1151	TATO	Santa Barbara	1985	0
SBMNH	1247	TATO	Santa Barbara	1986	0
MVZ	31615	RABO	San Luis Obispo	1939	0
MVZ	58422	RABO	San Luis Obispo	1953	0
MVZ	59660	RABO	San Luis Obispo	1953	0
MVZ	95868	HYRE	San Luis Obispo	1971	3
MVZ	107193	HYRE	San Luis Obispo	1972	0
MVZ	137354	HYRE	Santa Barbara	1976	3
MVZ	150009	HYRE	San Luis Obispo	1977	1
MVZ	227659	RACA	San Luis Obispo	1987	0
MVZ	240053	HYRE	Santa Barbara	1991	3
MVZ	27881	RACA	Los Angeles	1939	3
MVZ	32704	HYRE	Los Angeles	1940	0
MVZ	32705	HYRE	Los Angeles	1940	0
MVZ	32706	HYRE	Los Angeles	1940	0
MVZ	32708	HYRE	Los Angeles	1940	0
MVZ	32709	HYRE	Los Angeles	1940	0
MVZ	32710	HYRE	Los Angeles	1940	0
MVZ	32773	ANBO	Ventura	1940	0
MVZ	32774	HYCA	Ventura	1940	0

Appendix 2. Museum specimen data used for analysis. Reps positive = number of qPCR replicates (out of 3) in which Bd was detected. Species codes are available in Figure 3 of the main text.

Institution	Catalog #	Species	County	Year Collected	Reps Positive
MVZ	32775	HYCA	Ventura	1940	0
MVZ	33581	RADR	Santa Barbara	1940	0
MVZ	33725	HYCA	Ventura	1940	0
MVZ	33726	HYCA	Ventura	1940	0
MVZ	33727	HYCA	Ventura	1940	0
MVZ	33728	HYCA	Ventura	1940	0
MVZ	33729	HYCA	Ventura	1940	0
MVZ	33730	HYCA	Ventura	1940	0
MVZ	34596	HYRE	Santa Barbara	1940	1
MVZ	34597	HYRE	Santa Barbara	1940	0
MVZ	35217	ANCA	Santa Barbara	1940	0
MVZ	35218	ANCA	Santa Barbara	1940	0
MVZ	35219	HYRE	Santa Barbara	1940	0
MVZ	35220	HYRE	Santa Barbara	1940	0
MVZ	35221	HYRE	Santa Barbara	1940	0
MVZ	35364	ANBO	Ventura	1940	0
MVZ	35365	HYRE	Santa Barbara	1940	0
MVZ	35366	HYRE	Santa Barbara	1940	0
MVZ	57042	RADR	Los Angeles	1952	0
MVZ	59684	RADR	San Luis Obispo	1953	0
MVZ	61054	HYCA	Ventura	1954	0
MVZ	61055	HYCA	Ventura	1954	0
MVZ	61056	HYCA	Ventura	1954	0
MVZ	61057	HYCA	Ventura	1954	1
MVZ	61058	HYCA	Ventura	1954	0
MVZ	61059	HYCA	Ventura	1954	0
MVZ	61060	HYCA	Ventura	1954	0
MVZ	95869	HYRE	Santa Barbara	1971	1
MVZ	145257	ANBO	Santa Barbara	1977	0
MVZ	145258	ANBO	Santa Barbara	1977	0
MVZ	145259	ANBO	Santa Barbara	1977	0
MVZ	145260	ANBO	Santa Barbara	1977	0
MVZ	145261	ANBO	Santa Barbara	1977	0
MVZ	145262	ANBO	Santa Barbara	1977	0
MVZ	145263	ANBO	Santa Barbara	1977	0
MVZ	145264	ANBO	Santa Barbara	1977	0
MVZ	145265	ANBO	Santa Barbara	1977	3
MVZ	145266	ANBO	Santa Barbara	1977	0
MVZ	145267	ANBO	Santa Barbara	1977	0
MVZ	145268	ANBO	Santa Barbara	1977	0
MVZ	145269	ANBO	Santa Barbara	1977	0

Appendix 2. Museum specimen data used for analysis. Reps positive = number of qPCR replicates (out of 3) in which Bd was detected. Species codes are available in Figure 3 of the main text.

Institution	Catalog #	Species	County	Year Collected	Reps Positive
MVZ	145270	ANBO	Santa Barbara	1977	0
MVZ	145271	ANBO	Santa Barbara	1977	0
MVZ	145272	ANBO	Santa Barbara	1977	0
MVZ	35222	RABO	Santa Barbara	1940	0
MVZ	187306	RADR	Santa Barbara	1960	0
MVZ	187307	RADR	Santa Barbara	1960	0
MVZ	239695	ANBO	Santa Barbara	1992	2
MVZ	240615	ANBO	Ventura	1990	0
MVZ	33664	RABO	Ventura	1940	0
MVZ	33665	RABO	Ventura	1940	0
MVZ	33666	RABO	Ventura	1940	0
MVZ	33667	RABO	Ventura	1940	0
MVZ	33668	RABO	Ventura	1940	2
MVZ	33669	RABO	Ventura	1940	0
MVZ	33670	RABO	Ventura	1940	0
MVZ	33671	RABO	Ventura	1940	0
MVZ	33672	RABO	Ventura	1940	1
MVZ	33673	RABO	Ventura	1940	1
MVZ	42611	RABO	Ventura	1946	0
MVZ	54519	RABO	Ventura	1940	0
LACM	1360	HYRE	Los Angeles	1937	0
LACM	1656	RADR	San Luis Obispo	1946	0
LACM	1657	RADR	San Luis Obispo	1946	0
LACM	1658	RADR	San Luis Obispo	1946	0
LACM	13712	RABO	Los Angeles	1948	0
LACM	13713	RABO	Los Angeles	1948	0
LACM	13714	RABO	Los Angeles	1948	0
LACM	13715	RABO	Los Angeles	1948	0
LACM	13716	RABO	Los Angeles	1948	0
LACM	13717	RABO	Los Angeles	1948	1
LACM	13718	RABO	Los Angeles	1948	0
LACM	12303	HYCA	Santa Barbara	1965	0
LACM	12672	HYRE	Santa Barbara	1955	0
LACM	12678	HYRE	Santa Barbara	1965	0
LACM	12679	HYRE	Santa Barbara	1965	0
LACM	12681	HYRE	Santa Barbara	1965	0
LACM	12682	HYRE	Ventura	1953	0
LACM	12683	HYRE	Ventura	1953	0
LACM	12684	HYRE	Ventura	1954	0
LACM	12685	HYRE	Ventura	1954	0
LACM	12686	HYRE	Ventura	1963	0

Appendix 2. Museum specimen data used for analysis. Reps positive = number of qPCR replicates (out of 3) in which Bd was detected. Species codes are available in Figure 3 of the main text.

Institution	Catalog #	Species	County	Year Collected	Reps Positive
LACM	12687	HYRE	Ventura	1955	0
LACM	12688	HYRE	Ventura	1955	0
LACM	12690	HYRE	Ventura	1955	0
LACM	12691	HYRE	Ventura	1955	0
LACM	12692	HYRE	Ventura	1955	0
LACM	12693	HYRE	Ventura	1955	0
LACM	12694	HYRE	Ventura	1953	0
LACM	12695	HYRE	Ventura	1955	0
LACM	12696	HYRE	Ventura	1955	1
LACM	12697	HYRE	Ventura	1955	0
LACM	12698	HYRE	Ventura	1955	0
LACM	12699	HYRE	Ventura	1955	0
LACM	12700	HYRE	Ventura	1955	0
LACM	12701	HYRE	Ventura	1955	0
LACM	12702	HYRE	Ventura	1955	0
LACM	12703	HYRE	Ventura	1955	0
LACM	12704	HYRE	Ventura	1955	0
LACM	12705	HYRE	Ventura	1955	0
LACM	12706	HYRE	Ventura	1955	0
LACM	12707	HYRE	Ventura	1955	0
LACM	12708	HYRE	Ventura	1955	0
LACM	12709	HYRE	Ventura	1955	0
LACM	12710	HYRE	Ventura	1955	0
LACM	12711	HYRE	Ventura	1955	0
LACM	12712	HYRE	Ventura	1955	0
LACM	12713	HYRE	Ventura	1955	0
LACM	12714	HYRE	Ventura	1955	0
LACM	12715	HYRE	Ventura	1955	0
LACM	12716	HYRE	Ventura	1955	0
LACM	12718	HYRE	Ventura	1955	0
LACM	12720	HYRE	Ventura	1955	0
LACM	12721	HYRE	Ventura	1955	0
LACM	12722	HYRE	Ventura	1955	0
LACM	12723	HYRE	Ventura	1955	0
LACM	12724	HYRE	Ventura	1955	0
LACM	12790	HYRE	Los Angeles	1955	0
LACM	12798	HYRE	Los Angeles	1953	0
LACM	12800	HYRE	Los Angeles	1953	0
LACM	12808	HYRE	Los Angeles	1953	0
LACM	12811	HYRE	Los Angeles	1953	0
LACM	12815	HYRE	Los Angeles	1953	0

Appendix 2. Museum specimen data used for analysis. Reps positive = number of qPCR replicates (out of 3) in which Bd was detected. Species codes are available in Figure 3 of the main text.

Institution	Catalog #	Species	County	Year Collected	Reps Positive
LACM	12828	HYRE	Los Angeles	1965	0
LACM	12829	HYRE	Los Angeles	1965	0
LACM	12830	HYRE	Los Angeles	1963	0
LACM	12831	HYRE	Los Angeles	1963	0
LACM	12833	HYRE	Los Angeles	1963	0
LACM	12834	HYRE	Los Angeles	1964	0
LACM	12835	HYRE	Los Angeles	1964	0
LACM	12836	HYRE	Los Angeles	1964	0
LACM	12840	HYRE	Los Angeles	1942	0
LACM	12842	HYRE	Los Angeles	1965	0
LACM	12844	HYRE	Los Angeles	1964	0
LACM	12845	HYRE	Los Angeles	1964	0
LACM	12846	HYRE	Los Angeles	1964	0
LACM	12847	HYRE	Los Angeles	1964	0
LACM	12849	HYRE	Los Angeles	1964	0
LACM	12850	HYRE	Los Angeles	1964	0
LACM	12851	HYRE	Los Angeles	1964	0
LACM	12852	HYRE	Los Angeles	1964	0
LACM	12853	HYRE	Los Angeles	1964	0
LACM	12854	HYRE	Los Angeles	1965	0
LACM	12855	HYRE	Los Angeles	1954	0
LACM	12859	HYRE	Los Angeles	1954	0
LACM	12863	HYRE	Los Angeles	1964	0
LACM	12868	HYRE	Los Angeles	1953	0
LACM	12869	HYRE	Los Angeles	1953	0
LACM	12873	HYRE	Los Angeles	1963	0
LACM	12874	HYRE	Los Angeles	1963	0
LACM	12875	HYRE	Los Angeles	1963	0
LACM	12882	HYRE	Los Angeles	1964	0
LACM	12884	HYRE	Los Angeles	1954	0
LACM	12886	HYRE	Los Angeles	1964	0
LACM	12888	HYRE	Los Angeles	1964	0
LACM	12891	HYRE	Los Angeles	1963	0
LACM	12893	HYRE	Los Angeles	1963	0
LACM	12894	HYRE	Los Angeles	1964	0
LACM	12895	HYRE	Los Angeles	1964	0
LACM	12897	HYRE	Los Angeles	1964	0
LACM	12898	HYRE	Los Angeles	1955	0
LACM	12930	HYRE	Los Angeles	1955	0
LACM	12933	HYRE	Los Angeles	1955	0
LACM	12936	HYRE	Los Angeles	1955	0

Appendix 2. Museum specimen data used for analysis. Reps positive = number of qPCR replicates (out of 3) in which Bd was detected. Species codes are available in Figure 3 of the main text.

Institution	Catalog #	Species	County	Year Collected	Reps Positive
LACM	12939	HYRE	Los Angeles	1955	0
LACM	12940	HYRE	Los Angeles	1955	0
LACM	12945	HYRE	Los Angeles	1955	1
LACM	12947	HYRE	Los Angeles	1955	0
LACM	12950	HYRE	Los Angeles	1955	0
LACM	12951	HYRE	Los Angeles	1955	0
LACM	12955	HYRE	Los Angeles	1964	0
LACM	12964	HYRE	Los Angeles	1955	0
LACM	12965	HYRE	Los Angeles	1955	0
LACM	12987	HYRE	Los Angeles	1963	0
LACM	12996	HYRE	Los Angeles	1964	0
LACM	13005	HYRE	Santa Barbara	1963	0
LACM	13006	HYRE	Santa Barbara	1963	0
LACM	13007	HYRE	Santa Barbara	1963	0
LACM	13008	HYRE	Santa Barbara	1963	0
LACM	13009	HYRE	Los Angeles	1955	0
LACM	13196	HYCA	Ventura	1934	0
LACM	13197	HYCA	Ventura	1934	0
LACM	13198	HYCA	Ventura	1934	0
LACM	13204	HYCA	Ventura	1963	0
LACM	13205	HYCA	Ventura	1963	0
LACM	13207	HYCA	Ventura	1954	0
LACM	13209	HYCA	Ventura	1954	0
LACM	13213	HYCA	Ventura	1950	0
LACM	13215	HYCA	Ventura	1950	0
LACM	13216	HYCA	Ventura	1950	0
LACM	13218	HYCA	Ventura	1950	0
LACM	13219	HYCA	Ventura	1950	0
LACM	13221	HYCA	Ventura	1950	0
LACM	13223	HYCA	Ventura	1950	0
LACM	13225	HYCA	Ventura	1953	0
LACM	13454	RADR	Los Angeles	1947	0
LACM	13455	RADR	Los Angeles	1947	2
LACM	13457	RADR	Los Angeles	1963	2
LACM	13458	RADR	Los Angeles	1963	0
LACM	13459	RADR	Los Angeles	1963	0
LACM	13460	RADR	Los Angeles	1963	0
LACM	13461	RADR	Los Angeles	1964	1
LACM	13462	RADR	Los Angeles	1964	0
LACM	13463	RADR	Los Angeles	1964	0
LACM	13464	RADR	Los Angeles	1964	0

Appendix 2. Museum specimen data used for analysis. Reps positive = number of qPCR replicates (out of 3) in which Bd was detected. Species codes are available in Figure 3 of the main text.

Institution	Catalog #	Species	County	Year Collected	Reps Positive
LACM	13465	RADR	Los Angeles	1964	0
LACM	13466	RADR	Los Angeles	1964	0
LACM	13467	RADR	Los Angeles	1964	0
LACM	13468	RADR	Los Angeles	1963	0
LACM	13469	RADR	Los Angeles	1963	0
LACM	13470	RADR	Los Angeles	1954	0
LACM	13475	RADR	Los Angeles	1947	0
LACM	13496	RADR	Ventura	1954	2
LACM	13497	RADR	Ventura	1954	0
LACM	13498	RADR	Ventura	1950	1
LACM	13499	RADR	Ventura	1955	0
LACM	13500	RADR	Santa Barbara	1965	0
LACM	13501	RADR	Santa Barbara	1965	0
LACM	13502	RADR	Santa Barbara	1965	0
LACM	13503	RADR	Santa Barbara	1965	0
LACM	13531	RACA	Los Angeles	1963	0
LACM	13532	RACA	Los Angeles	1963	0
LACM	13539	RACA	Ventura	1963	0
LACM	13719	RABO	Los Angeles	1948	0
LACM	13720	RABO	Los Angeles	1948	0
LACM	13721	RABO	Los Angeles	1948	0
LACM	13722	RABO	Los Angeles	1948	0
LACM	13723	RABO	Los Angeles	1948	0
LACM	13724	RABO	Los Angeles	1948	1
LACM	13725	RABO	Los Angeles	1948	0
LACM	13726	RABO	Los Angeles	1948	0
LACM	13727	RABO	Los Angeles	1948	0
LACM	13728	RABO	Los Angeles	1950	0
LACM	13729	RABO	Los Angeles	1950	0
LACM	13730	RABO	Los Angeles	1950	0
LACM	13731	RABO	Los Angeles	1950	0
LACM	13732	RABO	Los Angeles	1950	0
LACM	13733	RABO	Los Angeles	1950	0
LACM	13734	RABO	Los Angeles	1950	1
LACM	13735	RABO	Los Angeles	1947	0
LACM	13738	RABO	Los Angeles	1950	0
LACM	13739	RABO	Los Angeles	1963	0
LACM	13740	RABO	Los Angeles	1963	0
LACM	13741	RABO	Los Angeles	1950	1
LACM	13742	RABO	Los Angeles	1950	0
LACM	13743	RABO	Los Angeles	1950	0

Appendix 2. Museum specimen data used for analysis. Reps positive = number of qPCR replicates (out of 3) in which Bd was detected. Species codes are available in Figure 3 of the main text.

Institution	Catalog #	Species	County	Year Collected	Reps Positive
LACM	13744	RABO	Los Angeles	1950	0
LACM	13745	RABO	Los Angeles	1950	0
LACM	13746	RABO	Los Angeles	1950	0
LACM	13764	RABO	Los Angeles	1955	0
LACM	13765	RABO	Los Angeles	1950	0
LACM	26591	RABO	Los Angeles	1959	0
LACM	26592	RABO	Los Angeles	1959	0
LACM	26593	RABO	Los Angeles	1960	0
LACM	26594	RABO	Los Angeles	1960	0
LACM	26595	RABO	Los Angeles	1960	0
LACM	26596	RABO	Los Angeles	1960	0
LACM	26597	RABO	Los Angeles	1966	0
LACM	91524	RABO	Los Angeles	1951	0
LACM	91525	RABO	Los Angeles	1951	0
LACM	91526	RABO	Los Angeles	1957	0
LACM	141854	RABO	Los Angeles	1944	1
LACM	141855	RABO	Los Angeles	1944	0
LACM	141856	RABO	Los Angeles	1944	0
LACM	141857	RABO	Los Angeles	1944	0
LACM	13767	RADR	Los Angeles	1956	0
LACM	13768	RADR	Los Angeles	1956	0
LACM	26454	HYRE	Los Angeles	1959	0
LACM	26456	HYRE	Los Angeles	1959	0
LACM	26457	HYRE	Los Angeles	1959	0
LACM	26459	HYRE	Los Angeles	1959	0
LACM	26460	HYRE	Los Angeles	1959	0
LACM	26462	HYRE	Los Angeles	1959	0
LACM	26464	HYRE	Los Angeles	1959	0
LACM	26469	HYRE	Los Angeles	1959	0
LACM	26470	HYRE	Los Angeles	1959	0
LACM	26485	HYRE	Los Angeles	1957	0
LACM	26486	HYRE	Los Angeles	1966	0
LACM	26487	HYRE	Los Angeles	1966	0
LACM	26489	HYRE	Los Angeles	1966	0
LACM	26491	HYRE	Los Angeles	1966	0
LACM	26492	HYRE	Los Angeles	1966	0
LACM	26493	HYRE	Los Angeles	1964	0
LACM	26582	RADR	Los Angeles	1960	0
LACM	26584	RADR	Los Angeles	1959	0
LACM	26585	RADR	Los Angeles	1959	0
LACM	26617	RACA	Los Angeles	1959	0

Appendix 2. Museum specimen data used for analysis. Reps positive = number of qPCR replicates (out of 3) in which Bd was detected. Species codes are available in Figure 3 of the main text.

Institution	Catalog #	Species	County	Year Collected	Reps Positive
LACM	26618	RACA	Los Angeles	1959	0
LACM	26619	RACA	Los Angeles	1959	0
LACM	26621	RACA	Los Angeles	1959	0
LACM	149145	RABO	San Luis Obispo	1999	0
LACM	35942	HYRE	Santa Barbara	1967	0
LACM	37657	HYRE	Los Angeles	1967	0
LACM	37659	HYRE	Los Angeles	1967	0
LACM	37660	HYRE	Los Angeles	1967	0
LACM	60428	HYRE	Ventura	1965	1
LACM	60429	HYRE	Ventura	1965	0
LACM	64217	HYCA	Ventura	1965	0
LACM	74408	RADR	Los Angeles	1965	0
LACM	74409	RADR	Los Angeles	1965	0
LACM	74410	RADR	Los Angeles	1965	0
LACM	74411	RADR	Los Angeles	1965	0
LACM	74412	RADR	Los Angeles	1950	0
LACM	74413	RADR	Los Angeles	1950	0
LACM	74681	HYRE	Los Angeles	1965	0
LACM	74682	HYRE	Los Angeles	1965	0
LACM	74688	HYRE	Los Angeles	1964	0
LACM	74689	HYRE	Los Angeles	1965	0
LACM	74690	HYRE	Los Angeles	1965	0
LACM	74691	HYRE	Los Angeles	1965	0
LACM	74695	HYRE	Los Angeles	1965	0
LACM	74696	HYRE	Los Angeles	1965	0
LACM	74697	HYRE	Los Angeles	1965	0
LACM	74703	HYRE	Los Angeles	1965	0
LACM	74778	HYRE	Los Angeles	1965	0
LACM	74788	RADR	Los Angeles	1965	0
LACM	74789	HYRE	Los Angeles	1965	0
LACM	74790	HYRE	Los Angeles	1965	0
LACM	74791	RADR	Ventura	1930	0
LACM	75214	HYRE	Los Angeles	1971	0
LACM	75215	HYRE	Los Angeles	1971	3
LACM	75695	HYRE	Ventura	1972	0
LACM	75696	HYRE	Ventura	1972	0
LACM	75698	HYRE	Ventura	1972	0
LACM	76274	RADR	Ventura	1968	3
LACM	76499	HYRE	Los Angeles	1966	0
LACM	76500	HYRE	Los Angeles	1966	0
LACM	89847	HYRE	Santa Barbara	1964	0

Appendix 2. Museum specimen data used for analysis. Reps positive = number of qPCR replicates (out of 3) in which Bd was detected. Species codes are available in Figure 3 of the main text.

Institution	Catalog #	Species	County	Year Collected	Reps Positive
LACM	89848	HYRE	Santa Barbara	1967	0
LACM	89849	HYRE	Santa Barbara	1967	0
LACM	89850	HYRE	Santa Barbara	1967	0
LACM	89851	HYRE	Santa Barbara	1967	0
LACM	89852	HYRE	Santa Barbara	1967	0
LACM	89853	HYRE	Santa Barbara	1967	0
LACM	89854	HYRE	Santa Barbara	1967	0
LACM	89855	HYRE	Santa Barbara	1967	0
LACM	89857	HYRE	Santa Barbara	1964	0
LACM	89868	HYRE	Santa Barbara	1964	0
LACM	89869	HYRE	Santa Barbara	1966	0
LACM	89876	HYRE	Ventura	1966	0
LACM	89877	HYRE	Ventura	1966	0
LACM	89878	HYRE	Ventura	1966	0
LACM	91109	RADR	Santa Barbara	1964	0
LACM	91110	RADR	Santa Barbara	1964	0
LACM	91141	RADR	Santa Barbara	1969	0
LACM	91535	RACA	Los Angeles	1964	0
LACM	91537	RACA	Los Angeles	1952	0
LACM	91539	RACA	Los Angeles	1955	0
LACM	91549	RACA	Los Angeles	1966	0
LACM	105871	HYCA	Santa Barbara	1969	0
LACM	105872	HYCA	Ventura	1970	1
LACM	105873	HYCA	Ventura	1970	0
LACM	105894	HYRE	Los Angeles	1972	3
LACM	105895	HYRE	Los Angeles	1972	3
LACM	105896	HYRE	Los Angeles	1972	1
LACM	105979	HYRE	San Luis Obispo	1968	0
LACM	105980	HYRE	Santa Barbara	1972	0
LACM	105981	HYRE	Santa Barbara	1972	0
LACM	105982	HYRE	Santa Barbara	1972	2
LACM	105987	HYRE	Ventura	1970	0
LACM	106030	RADR	San Luis Obispo	1968	0
LACM	106036	RACA	Los Angeles	1970	0
LACM	106037	RACA	Los Angeles	1970	0
LACM	126081	HYRE	Santa Barbara	1977	0
LACM	126085	RADR	Santa Barbara	1977	3
LACM	135325	RADR	Santa Barbara	1981	0
LACM	135326	RADR	Santa Barbara	1981	1
LACM	135327	RADR	Santa Barbara	1981	0
LACM	135328	RADR	Santa Barbara	1981	0

Appendix 2. Museum specimen data used for analysis. Reps positive = number of qPCR replicates (out of 3) in which Bd was detected. Species codes are available in Figure 3 of the main text.

Institution	Catalog #	Species	County	Year Collected	Reps Positive
LACM	135329	RADR	Santa Barbara	1981	0
LACM	135330	RADR	Santa Barbara	1981	0
LACM	135331	RADR	Santa Barbara	1981	0
LACM	135332	RADR	Santa Barbara	1981	0
LACM	135333	RADR	Santa Barbara	1981	0
LACM	135334	RADR	Santa Barbara	1981	2
LACM	135335	RADR	Santa Barbara	1981	4
LACM	135336	RADR	Santa Barbara	1981	0
LACM	135337	RADR	Santa Barbara	1981	0
LACM	135338	RADR	Santa Barbara	1981	4
LACM	135339	RADR	Santa Barbara	1981	4
LACM	135340	RADR	Santa Barbara	1981	0
LACM	135341	RADR	Santa Barbara	1981	3
LACM	135342	RADR	Santa Barbara	1981	0
LACM	135343	RADR	Santa Barbara	1981	0
LACM	135344	RADR	Santa Barbara	1981	3
LACM	137083	HYRE	Santa Barbara	1986	0
LACM	137804	HYRE	Ventura	1982	0
LACM	139127	RACA	San Luis Obispo	1991	3
LACM	141860	RACA	Los Angeles	1959	0
LACM	141861	RACA	Los Angeles	1959	0
LACM	141862	RACA	Los Angeles	1959	0
LACM	141863	RACA	Los Angeles	1959	0
LACM	141864	RACA	Los Angeles	1959	0
LACM	141865	RACA	Los Angeles	1959	0
LACM	141866	RACA	Los Angeles	1959	0
LACM	141867	RACA	Los Angeles	1959	0
LACM	141868	RACA	Los Angeles	1959	0
LACM	141869	RACA	Los Angeles	1943	0
LACM	141870	RACA	Los Angeles	1943	0
LACM	149087	HYCA	Los Angeles	2001	0
LACM	149088	HYCA	Los Angeles	2001	0
LACM	149094	HYCA	Los Angeles	2000	0
LACM	149095	HYCA	Los Angeles	2000	0
LACM	149097	HYCA	Los Angeles	2000	2
LACM	149140	RADR	San Luis Obispo	1999	3
LACM	149141	RADR	San Luis Obispo	2001	3
LACM	149142	RACA	San Luis Obispo	2001	3
LACM	149143	RACA	San Luis Obispo	2001	3
LACM	149144	RACA	San Luis Obispo	2001	3
LACM	149673	ANBO	Santa Barbara	2001	0

Appendix 2. Museum specimen data used for analysis. Reps positive = number of qPCR replicates (out of 3) in which Bd was detected. Species codes are available in Figure 3 of the main text.

Institution	Catalog #	Species	County	Year Collected	Reps Positive
LACM	150354	RADR	Ventura	2001	0
LACM	1956	RABO	Ventura	1930	0
LACM	13690	RABO	Ventura	1954	0
LACM	13691	RABO	Ventura	1963	0
LACM	13694	RABO	Ventura	1950	0
LACM	13695	RABO	Ventura	1950	0
LACM	13696	RABO	Ventura	1950	0
LACM	13697	RABO	Ventura	1950	1
LACM	13698	RABO	Ventura	1950	0
LACM	13699	RABO	Ventura	1954	0
LACM	13700	RABO	Ventura	1954	1
LACM	13701	RABO	Ventura	1954	0
LACM	13702	RABO	Ventura	1954	0
LACM	13703	RABO	Ventura	1954	2
LACM	13704	RABO	Ventura	1954	0
LACM	76268	RABO	Ventura	1968	0
LACM	76269	RABO	Ventura	1968	0
LACM	76270	RABO	Ventura	1968	0
LACM	76271	RABO	Ventura	1968	0
LACM	76272	RABO	Ventura	1968	0
LACM	76273	RABO	Ventura	1968	0
LACM	106062	RABO	Ventura	1970	0
CPP	116	RABO	Los Angeles	1966	3
CPP	683	RABO	Los Angeles	1968	0
CPP	850	RABO	Los Angeles	1970	3
CPP	1258	RABO	Los Angeles	1961	1
CPP	1263	RABO	Los Angeles	1969	0
CPP	1265	RABO	Los Angeles	1969	0
CPP	850B	RABO	Los Angeles	1970	3
CAS	7225	RABO	Los Angeles	1940	0
CAS	3601	ANBO	Los Angeles	1929	0
CAS	3602	ANBO	Los Angeles	1929	0
CAS	3603	ANBO	Los Angeles	1929	0
CAS	3604	ANBO	Los Angeles	1929	0
CAS	7226	RABO	Los Angeles	1940	0
CAS	7227	RABO	Los Angeles	1940	0
CAS	7228	RABO	Los Angeles	1940	0
CAS	7418	ANBO	Los Angeles	1940	0
CAS	10111	ANBO	Ventura	1949	0
CAS	10112	ANBO	Ventura	1949	0
CAS	10113	ANBO	Ventura	1949	0

Appendix 2. Museum specimen data used for analysis. Reps positive = number of qPCR replicates (out of 3) in which Bd was detected. Species codes are available in Figure 3 of the main text.

Institution	Catalog #	Species	County	Year Collected	Reps Positive
CAS	10114	ANBO	Ventura	1949	0
CAS	10115	ANBO	Ventura	1949	0
CAS	10116	ANBO	Ventura	1949	0
CAS	10117	ANBO	Ventura	1949	0
CAS	10119	ANBO	Ventura	1949	0
CAS	10120	ANBO	Ventura	1949	0
CAS	10121	ANBO	Ventura	1949	0
CAS	10122	ANBO	Ventura	1949	0
CAS	10123	ANBO	Ventura	1949	0
CAS	10129	ANCA	Ventura	1949	0
CAS	10130	ANCA	Ventura	1949	0
CAS	10131	ANCA	Ventura	1949	0
CAS	10132	ANCA	Ventura	1949	0
CAS	10133	ANCA	Ventura	1949	0
CAS	10134	ANCA	Ventura	1949	0
CAS	10135	ANCA	Ventura	1949	0
CAS	10136	ANCA	Ventura	1949	0
CAS	10137	ANCA	Ventura	1949	0
CAS	10138	ANCA	Ventura	1949	0
CAS	10139	ANCA	Ventura	1949	0
CAS	10140	ANCA	Ventura	1949	0
CAS	10141	ANCA	Ventura	1949	0
CAS	10142	ANCA	Ventura	1949	1
CAS	10143	ANCA	Ventura	1949	0
CAS	10144	ANCA	Ventura	1949	0
CAS	10145	ANCA	Ventura	1949	0
CAS	10146	ANCA	Ventura	1949	0
CAS	10148	ANCA	Ventura	1949	0
CAS	10149	ANCA	Ventura	1949	0
CAS	10150	ANCA	Ventura	1949	1
CAS	10152	ANCA	Ventura	1949	0
CAS	10200	HYRE	Ventura	1949	0
CAS	10201	HYRE	Ventura	1949	0
CAS	10212	RADR	Ventura	1949	0
CAS	10213	RADR	Ventura	1949	0
CAS	10214	RADR	Ventura	1949	0
CAS	10215	RADR	Ventura	1949	0
CAS	10216	RADR	Ventura	1949	0
CAS	10217	RADR	Ventura	1949	0
CAS	10218	RADR	Ventura	1949	0
CAS	10219	RADR	Ventura	1949	0

Appendix 2. Museum specimen data used for analysis. Reps positive = number of qPCR replicates (out of 3) in which Bd was detected. Species codes are available in Figure 3 of the main text.

Institution	Catalog #	Species	County	Year Collected	Reps Positive
CAS	10220	RADR	Ventura	1949	0
CAS	10633	HYRE	Los Angeles	1951	0
CAS	199438	RABO	Los Angeles	1969	0
CAS	199439	RABO	Los Angeles	1969	0
CAS	17655	TATO	Los Angeles	1956	0
CAS	17656	TATO	Los Angeles	1956	0
CAS	17657	TATO	Los Angeles	1956	0
CAS	17658	TATO	Los Angeles	1956	0
CAS	17659	TATO	Los Angeles	1956	0
CAS	17660	TATO	Los Angeles	1956	0
CAS	17661	TATO	Los Angeles	1956	0
CAS	27421	TATO	Los Angeles	1911	0
CAS	27422	TATO	Los Angeles	1911	0
CAS	27423	TATO	Los Angeles	1911	0
CAS	27424	TATO	Los Angeles	1911	0
CAS	27425	TATO	Los Angeles	1911	0
CAS	27426	TATO	Los Angeles	1911	0
CAS	27427	TATO	Los Angeles	1911	0
CAS	27428	TATO	Los Angeles	1911	0
CAS	27429	TATO	Los Angeles	1911	0
CAS	27430	TATO	Los Angeles	1911	0
CAS	27431	TATO	Los Angeles	1911	0
CAS	27432	TATO	Los Angeles	1911	0
CAS	39988	TATO	Los Angeles	1915	0
CAS	27374	ANBO	Los Angeles	1911	0
CAS	27375	ANBO	Los Angeles	1911	0
CAS	27410	RADR	Los Angeles	1911	0
CAS	27419	HYCA	Los Angeles	1911	0
CAS	39989	TATO	Los Angeles	1915	0
CAS	197451	TATO	Los Angeles	1993	3
CAS	197452	TATO	Los Angeles	1993	3
CAS	197453	TATO	Los Angeles	1993	0
CAS	197454	TATO	Los Angeles	1993	0
CAS	39177	RADR	Santa Barbara	1914	0
CAS	39865	ANBO	Los Angeles	1915	2
CAS	39866	ANBO	Los Angeles	1915	0
CAS	39867	ANBO	Los Angeles	1915	3
CAS	48999	RADR	Los Angeles	1919	0
CAS	49000	RADR	Los Angeles	1919	0
CAS	49033	HYCA	Los Angeles	1918	0
CAS	49034	HYCA	Los Angeles	1918	0

Appendix 2. Museum specimen data used for analysis. Reps positive = number of qPCR replicates (out of 3) in which Bd was detected. Species codes are available in Figure 3 of the main text.

Institution	Catalog #	Species	County	Year Collected	Reps Positive
CAS	49035	HYCA	Los Angeles	1918	0
CAS	49036	HYCA	Los Angeles	1918	0
CAS	49037	HYCA	Los Angeles	1918	0
CAS	49038	HYCA	Los Angeles	1918	0
CAS	49039	HYCA	Los Angeles	1918	0
CAS	49040	HYCA	Los Angeles	1918	0
CAS	49041	HYCA	Los Angeles	1916	0
CAS	49042	HYCA	Los Angeles	1916	0
CAS	49044	HYCA	Los Angeles	1916	0
CAS	49045	HYCA	Los Angeles	1916	0
CAS	49046	HYCA	Los Angeles	1916	0
CAS	49047	HYCA	Los Angeles	1916	0
CAS	49048	HYCA	Los Angeles	1916	0
CAS	49049	HYCA	Los Angeles	1916	0
CAS	50309	HYCA	Ventura	1921	0
CAS	50310	HYCA	Ventura	1921	0
CAS	50311	HYCA	Ventura	1921	0
CAS	50312	HYCA	Ventura	1921	0
CAS	50313	HYCA	Ventura	1921	0
CAS	50314	HYCA	Ventura	1921	0
CAS	50315	HYCA	Ventura	1921	0
CAS	50316	ANBO	Ventura	1921	0
CAS	50365	ANBO	Ventura	1921	0
CAS	50392	HYCA	Ventura	1921	0
CAS	50397	HYCA	Ventura	1921	0
CAS	50401	HYCA	Ventura	1921	0
CAS	50403	HYCA	Ventura	1921	0
CAS	50404	HYCA	Ventura	1921	0
CAS	50420	HYCA	Ventura	1921	0
CAS	50424	HYCA	Ventura	1921	0
CAS	50430	HYCA	Ventura	1921	0
CAS	50435	HYCA	Ventura	1921	0
CAS	50437	HYCA	Ventura	1921	0
CAS	50443	HYCA	Ventura	1921	0
CAS	50445	HYCA	Ventura	1921	0
CAS	50449	HYCA	Ventura	1921	0
CAS	50459	HYCA	Ventura	1921	0
CAS	50460	HYCA	Ventura	1921	0
CAS	63048	HYCA	Ventura	1927	0
CAS	63049	HYCA	Ventura	1927	2
CAS	63050	HYCA	Ventura	1927	0

Appendix 2. Museum specimen data used for analysis. Reps positive = number of qPCR replicates (out of 3) in which Bd was detected. Species codes are available in Figure 3 of the main text.

Institution	Catalog #	Species	County	Year Collected	Reps Positive
CAS	63051	HYCA	Ventura	1927	0
CAS	63052	HYCA	Ventura	1927	0
CAS	175693	ANBO	Los Angeles	1975	0
CAS	175694	ANBO	Los Angeles	1975	0
CAS	176387	ANBO	Ventura	1990	0
CAS	178491	RADR	Santa Barbara	1979	0
CAS	180836	TATO	Santa Barbara	1960	0
CAS	180837	TATO	Santa Barbara	1960	0
CAS	181039	ANBO	Santa Barbara	1960	0
CAS	181040	ANBO	Santa Barbara	1960	0
CAS	181041	ANBO	Santa Barbara	1960	0
CAS	181042	ANBO	Santa Barbara	1960	0
CAS	181043	ANBO	Santa Barbara	1961	0
CAS	181044	ANBO	Santa Barbara	1961	0
CAS	181045	ANBO	Santa Barbara	1961	0
CAS	181046	ANBO	Santa Barbara	1961	0
CAS	181047	ANBO	Santa Barbara	1961	0
CAS	181048	ANBO	Santa Barbara	1961	0
CAS	181049	ANBO	Santa Barbara	1961	0
CAS	181053	ANBO	Santa Barbara	1961	0
CAS	181054	ANBO	Santa Barbara	1961	0
CAS	181055	ANBO	Santa Barbara	1961	0
CAS	181057	ANBO	Santa Barbara	1961	0
CAS	181058	ANBO	Santa Barbara	1961	0
CAS	181059	ANBO	Santa Barbara	1961	0
CAS	181060	ANBO	Santa Barbara	1961	0
CAS	181061	ANBO	Santa Barbara	1961	0
CAS	181062	ANBO	Santa Barbara	1961	0
CAS	181064	ANBO	Santa Barbara	1961	0
CAS	181065	ANBO	Santa Barbara	1961	0
CAS	181066	ANBO	Santa Barbara	1961	0
CAS	181067	ANBO	Santa Barbara	1961	2
CAS	181068	ANBO	Santa Barbara	1961	0
CAS	181069	ANBO	Santa Barbara	1961	0
CAS	181071	ANBO	Santa Barbara	1961	0
CAS	181072	ANBO	Santa Barbara	1961	0
CAS	181073	ANBO	Santa Barbara	1961	0
CAS	181074	ANBO	Santa Barbara	1961	0
CAS	181075	ANBO	Santa Barbara	1961	0
CAS	181076	ANBO	Santa Barbara	1963	0
CAS	181077	ANBO	Santa Barbara	1966	0

Appendix 2. Museum specimen data used for analysis. Reps positive = number of qPCR replicates (out of 3) in which Bd was detected. Species codes are available in Figure 3 of the main text.

Institution	Catalog #	Species	County	Year Collected	Reps Positive
CAS	181078	ANBO	Santa Barbara	1966	0
CAS	181079	ANBO	Santa Barbara	1966	0
CAS	181080	ANBO	Santa Barbara	1966	0
CAS	181081	ANBO	Santa Barbara	1966	0
CAS	181082	ANBO	Santa Barbara	1966	0
CAS	181083	ANBO	Santa Barbara	1966	0
CAS	181084	ANBO	Santa Barbara	1966	0
CAS	181085	ANBO	Santa Barbara	1966	0
CAS	181086	ANBO	Santa Barbara	1966	0
CAS	181087	ANBO	Ventura	1958	0
CAS	181090	ANBO	Ventura	1960	0
CAS	181110	ANBO	Santa Barbara	1961	0
CAS	181111	ANBO	Santa Barbara	1961	0
CAS	181130	HYCA	Santa Barbara	1960	0
CAS	181131	HYCA	Santa Barbara	1960	0
CAS	181133	HYCA	Santa Barbara	1960	0
CAS	181134	HYCA	Santa Barbara	1960	0
CAS	181136	HYCA	Santa Barbara	1960	0
CAS	181137	HYCA	Santa Barbara	1960	0
CAS	181138	HYCA	Santa Barbara	1960	0
CAS	181139	HYCA	Santa Barbara	1960	0
CAS	181141	HYCA	Santa Barbara	1960	0
CAS	181142	HYCA	Santa Barbara	1960	0
CAS	181143	HYCA	Santa Barbara	1960	0
CAS	181145	HYCA	Santa Barbara	1960	0
CAS	181146	HYCA	Santa Barbara	1960	0
CAS	181147	HYCA	Santa Barbara	1960	0
CAS	181148	HYCA	Santa Barbara	1960	0
CAS	181153	HYCA	Santa Barbara	1966	0
CAS	181154	HYCA	Santa Barbara	1966	0
CAS	181155	HYCA	Santa Barbara	1966	0
CAS	181156	HYCA	Santa Barbara	1966	0
CAS	181157	HYCA	Santa Barbara	1966	0
CAS	181158	HYCA	Santa Barbara	1966	0
CAS	181159	HYCA	Santa Barbara	1966	1
CAS	181160	HYCA	Santa Barbara	1966	0
CAS	181161	HYCA	Santa Barbara	1966	0
CAS	181162	HYCA	Santa Barbara	1966	0
CAS	181163	HYCA	Santa Barbara	1966	0
CAS	181164	HYCA	Santa Barbara	1966	0
CAS	181165	HYCA	Ventura	1961	0

Appendix 2. Museum specimen data used for analysis. Reps positive = number of qPCR replicates (out of 3) in which Bd was detected. Species codes are available in Figure 3 of the main text.

Institution	Catalog #	Species	County	Year Collected	Reps Positive
CAS	181166	HYCA	Ventura	1961	0
CAS	181170	HYRE	Santa Barbara	1959	0
CAS	181171	HYRE	Santa Barbara	1959	0
CAS	181172	HYRE	Santa Barbara	1959	0
CAS	181173	HYRE	Santa Barbara	1959	0
CAS	181174	HYRE	Santa Barbara	1960	0
CAS	181175	HYRE	Santa Barbara	1960	0
CAS	181176	HYRE	Santa Barbara	1960	0
CAS	181177	HYRE	Santa Barbara	1960	0
CAS	181193	HYRE	Santa Barbara	1960	0
CAS	181195	HYRE	Santa Barbara	1960	0
CAS	181197	HYRE	Santa Barbara	1961	0
CAS	181198	HYRE	Santa Barbara	1961	0
CAS	181199	HYRE	Santa Barbara	1961	0
CAS	181200	HYRE	Santa Barbara	1961	0
CAS	181201	HYRE	Santa Barbara	1961	0
CAS	181205	HYRE	Santa Barbara	1961	0
CAS	181206	HYRE	Santa Barbara	1961	0
CAS	181207	HYRE	Santa Barbara	1961	0
CAS	181208	HYRE	Santa Barbara	1961	0
CAS	181209	HYRE	Santa Barbara	1961	0
CAS	181210	HYRE	Santa Barbara	1961	0
CAS	181213	HYRE	Santa Barbara	1961	0
CAS	181214	HYRE	Santa Barbara	1961	0
CAS	181215	HYRE	Santa Barbara	1964	0
CAS	181216	HYRE	Santa Barbara	1964	0
CAS	181217	HYRE	Santa Barbara	1964	0
CAS	181218	HYRE	Santa Barbara	1964	0
CAS	181219	HYRE	Santa Barbara	1959	0
CAS	181220	HYRE	Santa Barbara	1959	0
CAS	181221	HYRE	Santa Barbara	1959	0
CAS	181222	HYRE	Santa Barbara	1959	0
CAS	181223	RADR	Santa Barbara	1963	0
CAS	181224	RADR	Santa Barbara	1963	1
CAS	181225	RADR	Santa Barbara	1963	0
CAS	181226	RADR	Santa Barbara	1963	0
CAS	181227	RADR	Santa Barbara	1963	0
CAS	181228	RADR	Santa Barbara	1960	0
CAS	181229	RADR	Santa Barbara	1960	0
CAS	181230	RADR	Santa Barbara	1960	0
CAS	181231	RADR	Santa Barbara	1960	0

Appendix 2. Museum specimen data used for analysis. Reps positive = number of qPCR replicates (out of 3) in which Bd was detected. Species codes are available in Figure 3 of the main text.

Institution	Catalog #	Species	County	Year Collected	Reps Positive
CAS	181232	RADR	Santa Barbara	1960	0
CAS	181233	RADR	Santa Barbara	1960	0
CAS	181234	RADR	Santa Barbara	1960	0
CAS	181235	RADR	Santa Barbara	1960	0
CAS	181236	RADR	Santa Barbara	1960	0
CAS	181237	RADR	Santa Barbara	1960	0
CAS	181238	RADR	Santa Barbara	1960	0
CAS	181239	RADR	Santa Barbara	1960	0
CAS	181240	RADR	Santa Barbara	1960	0
CAS	181241	RADR	Santa Barbara	1960	0
CAS	181242	RADR	Santa Barbara	1960	0
CAS	181243	RADR	Santa Barbara	1960	0
CAS	181250	RADR	Santa Barbara	1961	0
CAS	181251	RADR	Santa Barbara	1961	0
CAS	181252	RADR	Santa Barbara	1961	0
CAS	181253	RADR	Santa Barbara	1961	0
CAS	181254	RADR	Santa Barbara	1961	0
CAS	181255	RADR	Santa Barbara	1961	0
CAS	181256	RADR	Santa Barbara	1961	0
CAS	181257	RADR	Santa Barbara	1961	0
CAS	181261	RADR	Santa Barbara	1962	0
CAS	181265	RADR	Santa Barbara	1966	0
CAS	181266	RADR	Santa Barbara	1966	0
CAS	181269	RABO	Santa Barbara	1960	0
CAS	181270	RABO	Santa Barbara	1960	0
CAS	181271	RABO	Santa Barbara	1960	0
CAS	187904	HYRE	San Luis Obispo	1988	0
CAS	188085	HYCA	Santa Barbara	1988	0
CAS	188086	HYCA	Santa Barbara	1988	0
CAS	188087	HYCA	Santa Barbara	1988	0
CAS	188088	HYCA	Santa Barbara	1988	0
CAS	190943	ANCA	Santa Barbara	1966	0
CAS	190944	ANCA	Santa Barbara	1966	2
CAS	190945	ANCA	Santa Barbara	1966	0
CAS	190946	ANCA	Santa Barbara	1966	0
CAS	190947	ANCA	Santa Barbara	1966	1
CAS	190948	ANCA	Santa Barbara	1966	0
CAS	190949	ANCA	Santa Barbara	1966	0
CAS	190950	ANCA	Santa Barbara	1966	0
CAS	190951	ANCA	Santa Barbara	1962	0
CAS	197401	HYCA	Los Angeles	1993	3

Appendix 2. Museum specimen data used for analysis. Reps positive = number of qPCR replicates (out of 3) in which Bd was detected. Species codes are available in Figure 3 of the main text.

Institution	Catalog #	Species	County	Year Collected	Reps Positive
CAS	197402	HYCA	Los Angeles	1993	3
CAS	197403	HYRE	Los Angeles	1993	0
CAS	197404	HYCA	Los Angeles	1993	3
CAS	197405	HYCA	Los Angeles	1993	0
CAS	197406	HYCA	Los Angeles	1993	3
CAS	197407	HYCA	Los Angeles	1993	0
CAS	197409	HYCA	Los Angeles	1993	0
CAS	197410	HYCA	Los Angeles	1993	1
CAS	197411	HYCA	Los Angeles	1993	3
CAS	197412	HYCA	Los Angeles	1993	0
CAS	197415	HYCA	Los Angeles	1994	0
CAS	197416	HYCA	Los Angeles	1994	3
CAS	197444	ANBO	Los Angeles	1993	0
CAS	197445	ANBO	Los Angeles	1993	3
CAS	180838	TATO	Santa Barbara	1960	0
CAS	180839	TATO	Santa Barbara	1960	0
CAS	180840	TATO	Santa Barbara	1960	0
CAS	180841	TATO	Santa Barbara	1960	0
CAS	197584	RADR	San Luis Obispo	1994	3
CAS	199428	ANBO	Los Angeles	1969	0
CAS	199429	ANBO	Los Angeles	1969	0
CAS	199430	ANBO	Los Angeles	1969	0
CAS	199431	ANBO	Los Angeles	1969	0
CAS	199432	ANBO	Los Angeles	1969	0
CAS	181272	RABO	Santa Barbara	1960	0
CAS	181273	RABO	Santa Barbara	1960	0
CAS	199458	HYRE	Los Angeles	1969	0
CAS	199459	HYRE	Los Angeles	1969	0
CAS	199460	HYRE	Los Angeles	1969	0
CAS	199461	HYRE	Los Angeles	1969	1
CAS	199462	HYCA	Los Angeles	1969	0
CAS	199463	HYCA	Los Angeles	1969	0
CAS	206482	HYCA	Ventura	1998	0
CAS	207434	ANBO	Los Angeles	1998	0
CAS	208506	ANBO	San Luis Obispo	1999	3
CAS	208507	ANBO	San Luis Obispo	1999	3
CAS	208510	HYRE	San Luis Obispo	1999	3
CAS	208511	HYRE	San Luis Obispo	1999	3
CAS	210394	RADR	San Luis Obispo	1998	3
CAS	210395	RADR	San Luis Obispo	1998	3
CAS	218286	RADR	Santa Barbara	1931	0

Appendix 2. Museum specimen data used for analysis. Reps positive = number of qPCR replicates (out of 3) in which Bd was detected. Species codes are available in Figure 3 of the main text.

Institution	Catalog #	Species	County	Year Collected	Reps Positive
CAS	233771	RACA	Los Angeles	2000	0
CAS	233772	RACA	Los Angeles	2000	0
CAS	233773	RACA	Los Angeles	2000	0
CAS	233774	RACA	Los Angeles	2000	3
CAS	244987	HYRE	Los Angeles	2008	3
CAS	244988	HYRE	Los Angeles	2008	3
CAS	181274	RABO	Santa Barbara	1960	0
CAS	181275	RABO	Santa Barbara	1961	0
CAS	10224	RABO	Ventura	1949	0
CAS	10225	RABO	Ventura	1949	0
CAS	10226	RABO	Ventura	1949	0
CAS	10227	RABO	Ventura	1949	0
CAS	10228	RABO	Ventura	1949	0
CAS	10229	RABO	Ventura	1949	0
CAS	39253	RABO	Ventura	1914	0
CAPO		RABO	San Luis Obispo	1958	0
CAPO		RABO	San Luis Obispo	1958	0
CAPO		RABO	San Luis Obispo	1959	0
CAPO		RABO	San Luis Obispo	1959	0
CAPO		RABO	San Luis Obispo	1959	0
CAPO		RABO	San Luis Obispo	1959	0
CAPO		RABO	San Luis Obispo	1959	0
CAPO		RABO	San Luis Obispo	1959	0
CAPO		RABO	San Luis Obispo	1959	0
CAPO		RABO	San Luis Obispo	1959	0
CAPO		RABO	San Luis Obispo	1959	0
CAPO		RABO	San Luis Obispo	1959	0
CAPO		RABO	San Luis Obispo	1959	0
CAPO		RABO	San Luis Obispo	1959	0
CAPO		RABO	San Luis Obispo	1959	0
CAPO		RABO	San Luis Obispo	1959	0
CAPO		RABO	San Luis Obispo	1959	0
CAPO		RABO	San Luis Obispo	1959	0
CAPO		RABO	San Luis Obispo	1959	0
CAPO		RABO	San Luis Obispo	1959	0
CAPO		RABO	San Luis Obispo	1959	0
CAPO		RABO	San Luis Obispo	1959	0
CAPO		RABO	San Luis Obispo	1959	0
CAPO		RABO	San Luis Obispo	1960	0
CAPO		RABO	San Luis Obispo	1960	0
CAPO		RABO	San Luis Obispo	1960	0

Appendix 2. Museum specimen data used for analysis. Reps positive = number of qPCR replicates (out of 3) in which Bd was detected. Species codes are available in Figure 3 of the main text.

Institution	Catalog #	Species	County	Year Collected	Reps Positive
CAPO		RABO	San Luis Obispo	1960	0
CAPO		RABO	San Luis Obispo	1960	0
CAPO		RABO	San Luis Obispo	1960	0
CAPO	H-00040	RADR	San Luis Obispo	1959	0
CAPO	H-00045	HYRE	San Luis Obispo	1958	0
CAPO	H-00102	RADR	San Luis Obispo	1958	0
CAPO	H-00127	RADR	San Luis Obispo	1958	0
CAPO	H-00128	RADR	San Luis Obispo	1958	0
CAPO	H-00149	RADR	San Luis Obispo	1958	0
CAPO	H-00150	RADR	San Luis Obispo	1958	0
CAPO	H-00151	RADR	San Luis Obispo	1958	0
CAPO	H-00159	HYRE	San Luis Obispo	1958	0
CAPO	H-00160	HYRE	San Luis Obispo	1958	0
CAPO	H-00164	HYRE	San Luis Obispo	1958	0
CAPO	H-00166	HYRE	San Luis Obispo	1958	0
CAPO	H-00175	RADR	San Luis Obispo	1958	0
CAPO	H-00197	RADR	San Luis Obispo	1958	1
CAPO	H-00198	RADR	San Luis Obispo	1958	0
CAPO	H-00199	RADR	San Luis Obispo	1958	0
CAPO	H-00203	HYRE	San Luis Obispo	1958	0
CAPO	H-00205	HYRE	San Luis Obispo	1958	0
CAPO	H-00206	HYRE	San Luis Obispo	1958	0
CAPO	H-00208	HYRE	San Luis Obispo	1958	0
CAPO	H-00209	HYRE	San Luis Obispo	1958	0
CAPO	H-00213	HYRE	San Luis Obispo	1958	0
CAPO	H-00215	HYRE	San Luis Obispo	1958	0
CAPO	H-00260	RADR	San Luis Obispo	1958	0
CAPO	H-00262	RADR	San Luis Obispo	1958	0
CAPO	H-00264	RADR	San Luis Obispo	1958	0
CAPO	H-00265	RADR	San Luis Obispo	1958	0
CAPO	H-00279	HYRE	San Luis Obispo	1958	0
CAPO	H-00280	HYRE	San Luis Obispo	1958	0
CAPO	H-00288	HYRE	San Luis Obispo	1958	0
CAPO	H-00294	RADR	San Luis Obispo	1958	0
CAPO	H-00295	RADR	San Luis Obispo	1958	0
CAPO	H-00296	RADR	San Luis Obispo	1958	0
CAPO	H-00297	RADR	San Luis Obispo	1958	0
CAPO	H-00299	RADR	San Luis Obispo	1958	0
CAPO	H-00300	RADR	San Luis Obispo	1958	0
CAPO	H-00302	RADR	San Luis Obispo	1958	0
CAPO	H-00303	RADR	San Luis Obispo	1958	0

Appendix 2. Museum specimen data used for analysis. Reps positive = number of qPCR replicates (out of 3) in which Bd was detected. Species codes are available in Figure 3 of the main text.

Institution	Catalog #	Species	County	Year Collected	Reps Positive
CAPO	H-00319	HYRE	San Luis Obispo	1958	0
CAPO	H-00320	HYRE	San Luis Obispo	1958	0
CAPO	H-00321	HYRE	San Luis Obispo	1958	0
CAPO	H-00817	RADR	San Luis Obispo	1958	0
CAPO		RADR	San Luis Obispo	1957	0
CAPO		RADR	San Luis Obispo	1958	0
CAPO		RADR	San Luis Obispo	1958	1
CAPO		RADR	San Luis Obispo	1958	0
CAPO		RADR	San Luis Obispo	1958	0
CAPO		RADR	San Luis Obispo	1958	0
CAPO		RADR	San Luis Obispo	1958	0
CAPO		RADR	San Luis Obispo	1958	0
CAPO		RADR	San Luis Obispo	1958	0
CAPO		RADR	San Luis Obispo	1958	0
CAPO		RADR	San Luis Obispo	1958	0
CAPO		RADR	San Luis Obispo	1958	1
CAPO		RADR	San Luis Obispo	1958	0
CAPO		RADR	San Luis Obispo	1958	0
CAPO		RADR	San Luis Obispo	1958	0
CAPO		RADR	San Luis Obispo	1958	0
CAPO		RADR	San Luis Obispo	1958	0
CAPO		RADR	San Luis Obispo	1958	0
CAPO		HYRE	San Luis Obispo	1958	0
CAPO		HYRE	San Luis Obispo	1958	0
CAPO		RADR	San Luis Obispo	1958	0
CAPO		RADR	San Luis Obispo	1958	0
CAPO		HYRE	Santa Barbara	1959	0
CAPO		HYRE	San Luis Obispo	1959	0
CAPO		RADR	San Luis Obispo	1959	0
CAPO		RADR	San Luis Obispo	1959	0
CAPO		RADR	San Luis Obispo	1959	0
CAPO		RADR	San Luis Obispo	1959	0
CAPO		RADR	San Luis Obispo	1959	0
CAPO		RADR	San Luis Obispo	1959	0
CAPO		RADR	San Luis Obispo	1959	0
CAPO		RADR	San Luis Obispo	1959	0
CAPO		RADR	San Luis Obispo	1959	0
CAPO		RADR	San Luis Obispo	1959	0
CAPO		RADR	San Luis Obispo	1959	0
CAPO		RADR	San Luis Obispo	1959	0
CAPO		HYRE	Santa Barbara	1959	0

Appendix 2. Museum specimen data used for analysis. Reps positive = number of qPCR replicates (out of 3) in which Bd was detected. Species codes are available in Figure 3 of the main text.

Institution	Catalog #	Species	County	Year Collected	Reps Positive
CAPO		RADR	San Luis Obispo	1959	0
CAPO		RADR	San Luis Obispo	1959	0
CAPO		RADR	San Luis Obispo	1959	0
CAPO		RADR	San Luis Obispo	1959	0
CAPO		RADR	San Luis Obispo	1959	0
CAPO		RADR	San Luis Obispo	1959	0
CAPO		RADR	San Luis Obispo	1959	0
CAPO		RADR	San Luis Obispo	1959	0
CAPO		RADR	San Luis Obispo	1959	0
CAPO		RADR	San Luis Obispo	1959	1
CAPO		RADR	San Luis Obispo	1959	0
CAPO		RADR	San Luis Obispo	1959	0
CAPO		RADR	San Luis Obispo	1959	0
CAPO		RADR	San Luis Obispo	1959	0
CAPO		RADR	San Luis Obispo	1959	0
CAPO		RADR	San Luis Obispo	1959	0
CAPO		RADR	San Luis Obispo	1959	0
CAPO		RADR	San Luis Obispo	1959	0
CAPO		RADR	San Luis Obispo	1960	0
CAPO		RADR	San Luis Obispo	1960	0
CAPO		RADR	San Luis Obispo	1960	0
CAPO		RADR	San Luis Obispo	1960	0
CAPO		RADR	Santa Barbara	1960	0
CAPO		RADR	San Luis Obispo	1960	0
CAPO		RADR	San Luis Obispo	1961	0
CAPO		HYRE	San Luis Obispo	1963	0
CAPO		RADR	San Luis Obispo	1965	0
CAPO		HYRE	San Luis Obispo	1965	0
CAPO		HYRE	San Luis Obispo	1965	0
CAPO		RADR	San Luis Obispo	1965	0
CAPO		HYRE	San Luis Obispo	1965	0
CAPO		HYRE	San Luis Obispo	1967	0
CAPO		HYRE	San Luis Obispo	1967	0
CAPO		HYRE	San Luis Obispo	1967	0
CAPO		RACA	San Luis Obispo	1967	0
CAPO		RADR	San Luis Obispo	1967	0
CAPO		HYRE	San Luis Obispo	1967	0
CAPO		HYRE	San Luis Obispo	1967	0
CAPO		HYRE	San Luis Obispo	1967	0
CAPO		HYRE	San Luis Obispo	1967	0
CAPO		HYRE	San Luis Obispo	1967	0

Appendix 2. Museum specimen data used for analysis. Reps positive = number of qPCR replicates (out of 3) in which Bd was detected. Species codes are available in Figure 3 of the main text.

Institution	Catalog #	Species	County	Year Collected	Reps Positive
CAPO		HYRE	San Luis Obispo	1967	0
CAPO		HYRE	San Luis Obispo	1967	0
CAPO		HYRE	San Luis Obispo	1967	0
